# Characterisation of putative novel tick viruses and zoonotic risk prediction

**DOI:** 10.1002/ece3.10814

**Published:** 2024-01-21

**Authors:** Yuting Lin, David J. Pascall

**Affiliations:** ^1^ MRC Biostatistics Unit University of Cambridge Cambridge UK; ^2^ Royal Veterinary College University of London London UK

**Keywords:** tick viruses, virus discovery, zoonotic prediction

## Abstract

Tick‐associated viruses remain a substantial zoonotic risk worldwide, so knowledge of the diversity of tick viruses has potential health consequences. Despite their importance, large amounts of sequences in public data sets from tick meta‐genomic and ‐transcriptomic projects remain unannotated, sequence data that could contain undocumented viruses. Through data mining and bioinformatic analysis of more than 37,800 public meta‐genomic and ‐transcriptomic data sets, we found 83 unannotated contigs exhibiting high identity with known tick viruses. These putative viral contigs were classified into three RNA viral families (Alphatetraviridae, Orthomyxoviridae and Chuviridae) and one DNA viral family (Asfarviridae). After manual checking of quality and dissimilarity towards other sequences in the data set, these 83 contigs were reduced to five contigs in the Alphatetraviridae from four putative viruses, four in the Orthomyxoviridae from two putative viruses and one in the Chuviridae which clustered with known tick‐associated viruses, forming a separate clade within the viral families. We further attempted to assess which previously known tick viruses likely represent zoonotic risks and thus deserve further investigation. We ranked the human infection potential of 133 known tick‐associated viruses using a genome composition‐based machine learning model. We found five high‐risk tick‐associated viruses (Langat virus, Lonestar tick chuvirus 1, Grotenhout virus, Taggert virus and Johnston Atoll virus) that have not been known to infect human and two viral families (Nairoviridae and Phenuiviridae) that contain a large proportion of potential zoonotic tick‐associated viruses. This adds to the knowledge of tick virus diversity and highlights the importance of surveillance of newly emerging tick‐associated diseases.

## Graphical abstract

Tick‐borne viruses remain a substantial zoonotic risk worldwide, so knowledge of the diversity of tick viruses has potential health consequences. Through data mining and bioinformatic analyses of more than 37,800 public meta‐genomic and ‐transcriptomic data sets, we found five putative novel Alphatetra‐like viruses, four putative novel Orthomyxo‐like viruses and one Chu‐like virus which clustered with known tick‐borne viruses. We also assessed which previously known tick viruses likely represent zoonotic risks and thus deserve further investigation, finding two viral families (Nairoviridae and Phenuiviridae) that contain a large proportion of potential zoonotic tick‐borne viruses.

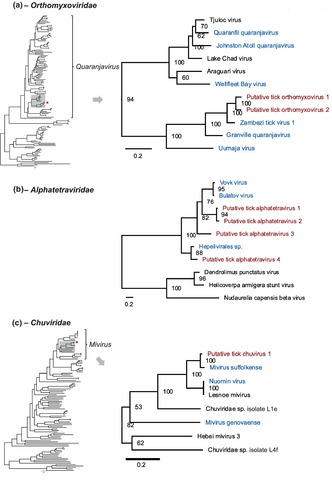

## 1 INTRODUCTION

The role of ticks in the transmission of viruses has been known for over 100 years (Stockman, 1918) with ticks being second only to mosquitoes as vectors of pathogens to humans and the primary vector of pathogens to livestock, wildlife and companion animals (Mansfield et al., 2017). Nonetheless, new tick viruses are still being described (Liu et al., 2022). Two recent examples are Yezo virus identified in Japan (Kodama et al., 2021) and Songling virus identified in China (Ma et al., 2021), which are both orthonairoviruses in the family Nairoviridae and have both been associated with acute human febrile disease.

Over the last two decades, meta‐genomics and ‐transcriptomics tools have revolutionised virus discovery. These approaches employ high‐throughput sequencing technologies to capture total viral communities in a relatively unbiased manner (Mokili et al., 2012; Tokarz & Lipkin, 2021) and have facilitated studies of the tick virome that resulted in the discovery and characterisation of hundreds of new tick‐associated viruses globally (Bouquet et al., 2017; Chandra et al., 2021; Garrison et al., 2020; Harvey et al., 2019; Li et al., 2015; Pettersson et al., 2017; \Shi et al., 2016, 2021; Tokarz et al., 2018; Xu et al., 2021). For many of these newly discovered viruses, we possess only a single sequence (which may or may not constitute a complete genome), with a lack of associated phenotypic data, available cultures or isolates. Despite these constraints, the data derived from these discoveries holds immense potential, especially in its possibility to assess human infection potential, thereby enabling the utilisation of this information for public health benefits, as explored further below.

In the realm of zoonotic risk assessment, conventional models often rely on the presence of zoonotic viruses within the same viral family or their evolutionary relatedness to known zoonoses (Bergner et al., 2021). These models rely on phenotypic information commonly unavailable for novel viruses or of insufficient resolution to estimate different levels of zoonotic potential for closely related viruses (Grange et al., 2021; Olival et al., 2017; Pulliam & Dushoff, 2009). More recent models, which are based on the genomic features of viruses and do not exclusively hinge on phylogeny or taxonomy, circumvent this issue. Such models use genomic features that may individually contain weak signals but can be combined and exploited via machine learning algorithms (Babayan et al., 2018; Bartoszewicz et al., 2021; Mollentze et al., 2021; Zhang et al., 2019). Particularly, such models, solely constructed from genomic data, excel in evaluating newly discovered viruses that lack other sources of information. However, common drawbacks of this approach are that such models typically require large amounts of training data, struggle to generalise beyond the distribution of the training dataset, and are constrained by the prerequisite for complete viral genomes to ensure accurate assessment (Bartoszewicz et al., 2021; Bergner et al., 2021; Mollentze et al., 2021).

In this study, we aimed to describe novel viruses that may have been overlooked by previous tick meta‐genomic and ‐transcriptomic studies, and assess the zoonotic risk posed by tick virus diversity as it is currently understood. Thus, we searched publicly available tick sequence data for signs of viruses misidentified as tick sequences and evaluated the zoonotic risk of these and other tick viruses to find those which deserve further investigation.

## 2 MATERIALS AND METHODS

### 2.1 Data sets

We collected tick virus data from reports published prior to December 2021 and constructed a tick virus data set that contained 261 tick‐associated viruses from 23 viral families (34 genera) and 12 putative tick‐associated viruses sampled from tick pools. Tick‐associated viruses with unknown tick vectors (*N* = 13) or sequences unavailable (*N* = 13) were excluded from the analysis. This resulted in 235 viruses in the final tick virus database (including both zoonotic viruses and arthropod‐specific viruses found in ticks); three of them (Amblyomma dissimile mivirus, Nuomin virus, and Granville quaranjavirus) were added later and not included in the database for virus discovery. Among the 235 tick‐associated viruses, more than 92% of viruses were RNA viruses, while few other viral sequences were identified (2.2% DNA viruses and 5.6% viruses with unknown genome composition). Among the RNA viruses, most sequences were negative‐sense (−) single‐stranded RNA (ssRNA) viruses (63.0%), followed by positive‐sense (+) single‐stranded RNA (ssRNA) (20.8%), double‐stranded RNA (dsRNA) (8.5%) and unclassified RNA (5.6%) viruses, depending on version 2020 of the International Committee on Taxonomy of Viruses (ICTV) master species list (https://talk.ictvonline.org/files/master‐species‐lists/). We labelled each virus as being capable of infecting humans or not known to infect humans using published reports as ground truth. In all cases, only viruses detected in humans by either PCR or sequencing were considered to have proven ability to infect humans. See the summary table of currently known tick‐associated viruses in Table A1 in Appendix 1.

### 2.2 Viral identification and annotation

We used representative genomic sequences of the 232 tick‐associated viruses as our reference database, with arthropod‐specific genome segments of segmented viruses concatenated into one. Using the ‘blastn_vdb’ (corresponding to nucleotide BLAST) executable in the BLAST+ v2.13.0 (26–28), we searched the Sequence Read Archive (SRA), Transcriptome Shotgun Assembly (TSA) and Whole Genome Shotgun (WGS) data sets that derived from ticks and subordinate taxa directly with an expectation cut‐off of 0.05. We also performed a ‘tBLASTx’ search for the TSA database to find distant relationships between nucleotide sequences; ‘tBLASTx’ searches were not used for the SRA and WGS data sets due to computational limitations. This resulted in 1328 TSA, 19,990 SRA and 1861 WGS sequences after removing duplicate viral names. The GenBank accession numbers of the candidate sequences are provided at https://github.com/ytlin2021/TickVirus.git.

**FIGURE 1:**
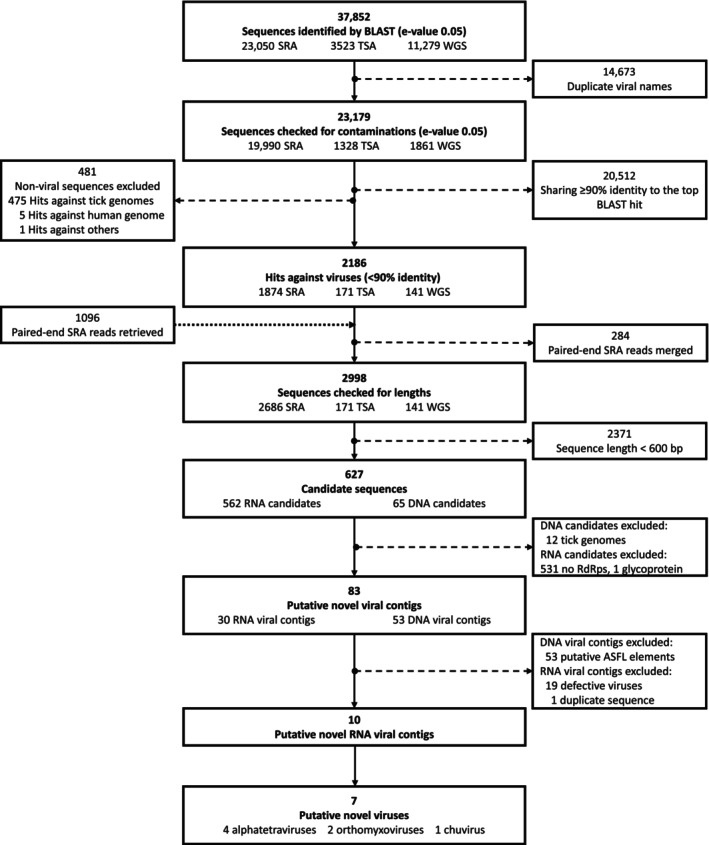
The workflow of identification of novel tick‐associated viral contigs. Through BLAST search and curation, 53 DNA sequences (identified as African swine fever‐like elements) and 30 RNA sequences (putative viruses in the family Alphatetraviridae, Orthomyxoviridae and Chuviridae) from a collection of tick meta‐genomic and ‐transcriptomic data sets (3 March 2022) were identified as putative novel viral contigs. Ten putative RNA viral contigs were further analysed after filtering. ASFL, African swine fever like; E‐value, Expectation value; SRA, Sequence Read Archive; TSA, Transcriptome Shotgun Assembly; WGS, Whole Genome Shotgun.

**FIGURE A1:**
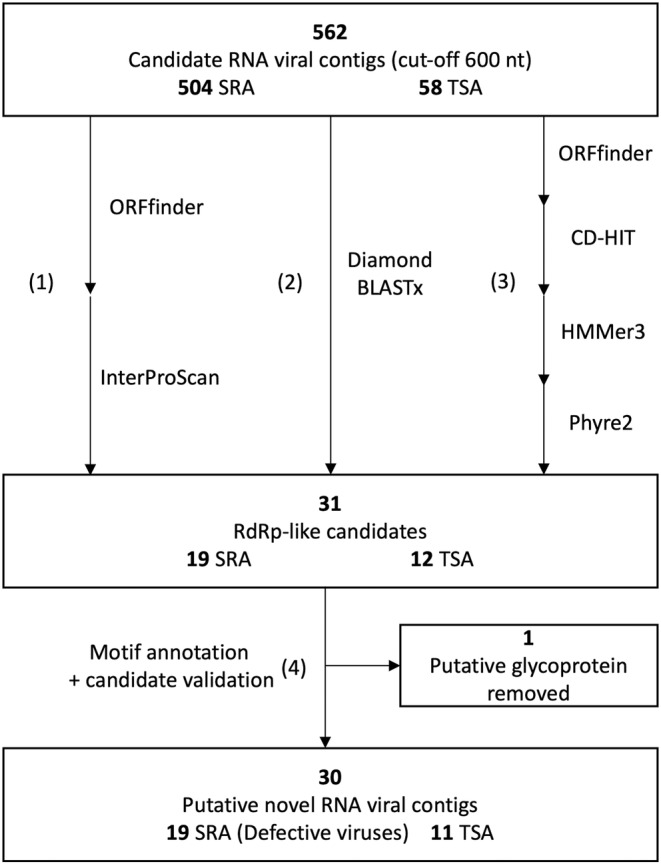
Workflow of detecting viral RNA‐dependent RNA polymerase sequences. (1) ORFs were translated using ORFfinder (cut‐off 200 amino acid). The translated sequences were predicted using the InterProScan‐5.55–88.0 (‐‐minsize 600; ‐goterms). (2) BLASTx using Diamond v2.0.14.152 against both non‐redundant protein database and RdRp‐scan database (‐e 1e‐5 ‐‐min‐orf 600 ‐‐very‐sensitive). (3) ORFs were translated using ORFfinder (cut‐off 200 amino acid). Redundant sequences were then removed using CD‐HIT v4.6.8 at 90% level of identity (‐c 0.98 sequence identity threshold). Remaining sequences were scanned using HMMer3 v3.2.1 against RdRp HMM profile database (full sequence e‐value 1e‐06). HMM‐RdRp hits were compared against PDB using Phyre2 server. In total, 31 RdRp‐like candidates from SRA and TSA databases were found using RdRp‐scan and InterProScan after removing duplicates. (4) RdRp motifs were mapped to candidate sequences using Geneious v2022.1. RdRp‐scan database, RdRp HMM profile database, RdRp motifs database and RdRp key word lists were obtained from (Charon et al., 2022) (https://github.com/JustineCharon/RdRp‐scan).

We further filtered candidate sequences according to the pipeline modified from Webster et al. (2015). The cut‐offs used in each step are seen in Figure 1. To confirm final RNA candidates, we checked the presence of RNA‐dependent RNA polymerase (RdRp), an essential protein encoded in the genomes of all replicating RNA viruses without a DNA stage except deltaviruses, and not present in the genome of the eukaryotic or prokaryotic cell. We adopted a combined analysis approach to predict RdRps using (1) a general protein function prediction software package InterProScan v5.55‐88.0 (Jones et al., 2014) and (2) a new open bioinformatics toolkit—RdRp‐scan—that allowed the detection of divergent viral RdRp sequences (Charon et al., 2022). After excluding candidate sequences shorter than 600 nucleotides (nt) in length, we proceeded to identify RdRp‐like candidates within the translated sequences. Then, we annotated the conserved RdRp motifs manually based on the RdRp motif database in RdRp‐scan, and confirmed final RNA viral candidates. The workflow of detecting viral RdRp sequences and the thresholds used in each step can be seen in Figure A1 in Appendix 1. The DNA candidates were not analysed further; see Section 3.

Protein functions of putative novel viral contigs were annotated using InterProScan (Jones et al., 2014) and ‘BLASTx’ (Altschul et al., 1990, 1997; Camacho et al., 2009). Open Reading Frames (ORFs) were predicted using ORF Finder tool in NCBI (Wheeler et al., 2008) with a 150 nt length cut‐off. InterProScan searched for protein function annotations in its available databases, including the Gene Ontology (Ashburner et al., 2000; Hennig et al., 2003) and Pfam (Mistry et al., 2021; Punta et al., 2012) databases. At the same time, ‘BLASTx’ were used to search against the non‐redundant protein database with an expectation cut‐off of 1e‐5 following Charon et al. (2022).

### 2.3 Phylogenetics

We searched for close and distant relatives of the identified novel viral contigs in the ‘viruses’ (NCBI taxa ID: 10239) non‐redundant nucleotide and protein databases by performing ‘BLASTn’ and ‘BLASTx’ separately with an expectation cut‐off of 0.005 (Altschul et al., 1990, 1997; Camacho et al., 2009). For each contig with BLAST hits, the phylogenetic hierarchy of the best hits (top 10 hits from ‘BLASTx’) was traversed upwards to identify the lowest taxonomic classification displaying a 75% majority taxonomic agreement following Webster et al. (2015). If the best hits were unclassified viruses, the upper order was identified, and the protein sequences were aligned in each family under the order to determine the classification. This allowed putative novel viral contigs in the study to be classified into three RNA viral families (Alphatetraviridae, Orthomyxoviridae and Chuviridae) and one DNA viral family (Asfarviridae). For the RNA viruses, all virus species in the same family, as well as related unassigned viruses, were aligned and used to construct phylogenetic trees.

Phylogeny of RNA viruses was inferred on the basis of RNA‐dependent RNA polymerase protein sequences. We aligned viral RdRp protein sequences using T‐Coffee (Notredame et al., 2000) on the EMBL‐EBI server (Madeira et al., 2022), poorly aligned sequences were removed and the alignment was reperformed. Alignments were then trimmed to the well‐aligned region by eye.

Tree estimation was performed in PhyML (Guindon et al., 2010) using the best fitting model as assessed AICc in model generator (Keane et al., 2006) (Orthomyxoviridae and Alphatetraviridae, LG + G + F; Chuviridae, LG + G). The Alphatetraviridae and Chuviridae trees were run using PhyML version 3:3.3.20170530 + dfsg‐2. The Orthomyxoviridae tree was run using the Monpellier Bioinformatics Platform PhyML server. All trees were run using the best of SPR and NNI searches, four gamma categories with 104 bootstrap replicates and at least 10 random starts.

### 2.4 Zoonotic risk analysis

The zoonotic risk (precisely, probability of human infection given biologically relevant exposure) for 133 tick‐associated viruses with complete genome sequences were evaluated using the best performing model from Mollentze et al. (2021), referred to here as genome composition‐based (GCB), which was trained on a range of viral genome features (i.e. codon usage biases, amino acid biases and dinucleotide biases), calculated directly from viral genome or based on the similarity of viral genome composition to human gene transcripts (Mollentze et al., 2021). A representative genome was selected for each virus, giving preference to sequences from NCBI Reference Sequence Database (RefSeq) (www.ncbi.nlm.nih.gov/refseq/) wherever possible. RefSeq sequences that had annotation issues or were not judged to be representative of the naturally circulating virus were replaced with alternative genomes. Following Mollentze et al. (2021), the predicted probabilities from the GCB model were used to categorise viruses into four priority categories based on the overlap of confidence intervals (CIs) with the 0.293 cut‐off from above (low: entire 95% CI of predicted probability ≤ cut‐off; medium: mean prediction ≤ cut‐off, but CI crosses it; high: mean prediction > cut‐off, but CI crosses it; and very high: entire CI > cut‐off). Since the current GCB model was trained for viruses with complete sequences, 99 tick viruses with only partial genomes available were excluded from the analysis. To characterise the zoonotic potential of the identified novel viral contigs, we did qualitative analysis by evaluating the published complete genomes of known viruses most similar to these incomplete viral sequences as determined by a nucleotide BLAST against GenBank.

## 3 RESULTS

### 3.1 Identification of putative novel viruses associated with ticks

We examined contigs that showed nucleotide similarity (<90% identity) to previously characterised tick virus sequences from an extensive collection of tick meta‐genomic and ‐transcriptomic data sets (23,050 SRA, 3523 TSA and 11,279 WGS sequences; collected on 3 March 2022). Following Fauquet and Stanley (2005), contigs exhibiting greater than 10% difference in nucleotide identity are considered putative novel viruses, and those exhibiting less than 10% difference are strains of known viruses. The pipeline of discovering novel tick‐associated viral contigs can be found in Figure 1. For more information on the tick virus database and tick meta‐genomic and ‐transcriptomic data sets, see Section 2.

In total, we identified 83 putative novel viral contigs, including 53 double‐stranded DNA viral contigs, five positive‐sense and 25 negative‐sense RNA viral contigs (Tables A2, A3, A4 in Appendix 1). The 53 DNA viral contigs belong to the family Asfarviridae, with homology to African swine fever virus. These African swine fever virus‐like contigs were found in *Ornithodoros porcinus* and *Ornithodoros moubata* ticks (Table A4 in Appendix 1), and all but two of these contigs derived from a study that had previously reported the integration of African swine fever like elements into the genomes of these soft tick species (Forth et al., 2020), so they will not be discussed further. The five positive‐sense RNA viral contigs belong to the family Alphatetraviridae which includes arthropod‐specific viruses found in ticks. Of the negative‐sense RNA viral contigs, five belong to the family Orthomyxoviridae, and 20 belong to the family Chuviridae, including 19 chuviruses believed to be replication defective from tick SRA projects. After filtering duplicate sequences (GenBank IDs: HACP01027211.1 and HACW01024387.1), we obtained 10 putative RNA viral contigs ranging from 728 to 5496 bp (Table 1) from which we described seven putative novel viruses. The putative novel viruses found in this study were named after their host order and related viral family, followed by a number (e.g. Putative tick alphatetravirus 1). All sequences identified in this study can be found on GenBank (see accession numbers in Table 1 and Tables A2, A3, A4 in Appendix 1).

**TABLE 1:** The putative novel tick viruses found after bioinformatic checking.

Putative novel viruses	Tick species	Geographical location	Provisional family	Genome structure	Closest BLASTx match (% amino acid identity)	GenBank ID	Source data
Putative tick orthomyxovirus 1	*Rhipicephalus sanguineus*	Italy	Orthomyxoviridae	ssRNA(−)	Zambezi tick virus 1 (89)	BK062912.1	HACP01027211.1, HACW01024387.1, HACP01022575.1, HACW01018819.1
Putative tick orthomyxovirus 2	*Rhipicephalus haemaphysaloides*	China	Orthomyxoviridae	ssRNA(−)	Zambezi tick virus 1 (89)	BK062910.1	GIJA01018702.1
Putative tick alphatetravirus 1	*Ixodes ricinus*	China	Alphatetraviridae	ssRNA(+)	Bulatov virus (44)	BK062906.1	GIXL01013119.1
Putative tick alphatetravirus 2	*Ixodes persulcatus*	USA	Alphatetraviridae	ssRNA(+)	Vovk virus (60)	BK062905.1	GBXQ01012426.1
Putative tick alphatetravirus 3	*Ornithodoros moubata*	Spain	Alphatetraviridae	ssRNA(+)	Bulatov virus (40)	BK062908.1	GIXP02005464.1, GFJQ02007921.1
Putative tick alphatetravirus 4	*Rhipicephalus microplus*	USA	Alphatetraviridae	ssRNA(+)	Hepelivirales sp. (80)	BK062911.1	GEMR01004364.1
Putative tick chuvirus 1	*Ixodes scapularis*	USA	Chuviridae	ssRNA(−)	Nuomin virus (89)	BK062907.1	GGIX01201876.1

*Note*: Results were obtained on 27 November 2022.

Abbreviations: PB1, polymerase basic protein 1; RdRp, RNA‐dependent RNA polymerase.

#### 3.1.1 Putative novel tick orthomyxoviruses

**FIGURE 2:**
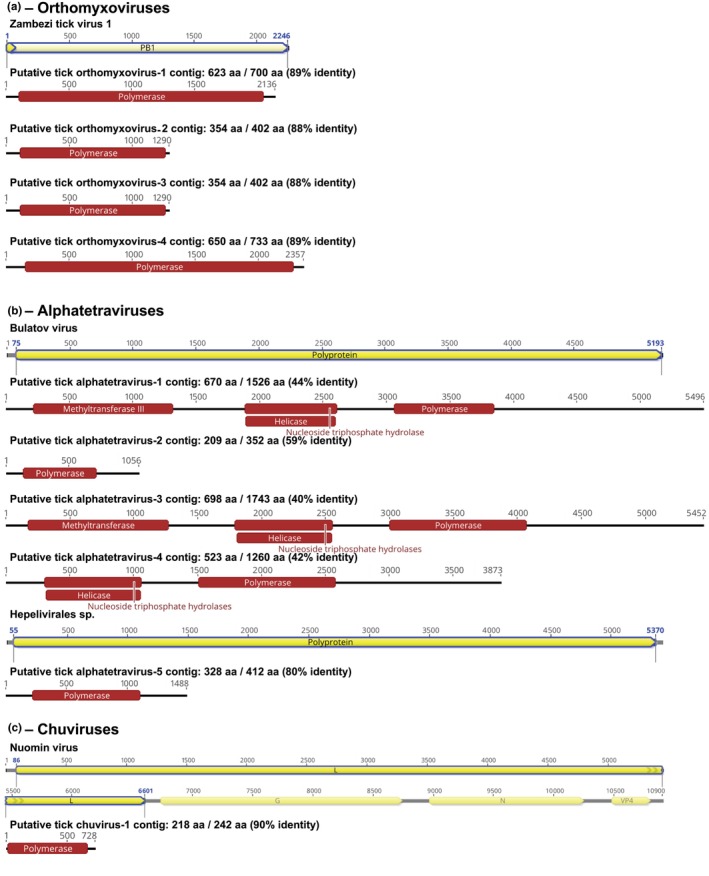
Illustrations of predicted protein positions in putative novel viral contigs. Protein functions of putative novel viral contigs were annotated using InterProScan (Jones et al., 2014) and ‘BLASTx’ (Altschul et al., 1990, 1997; Camacho et al., 2009). Shown per cent of amino acid identity (%) between the novel viral contigs and their closest BLASTx match.

**FIGURE 3:**
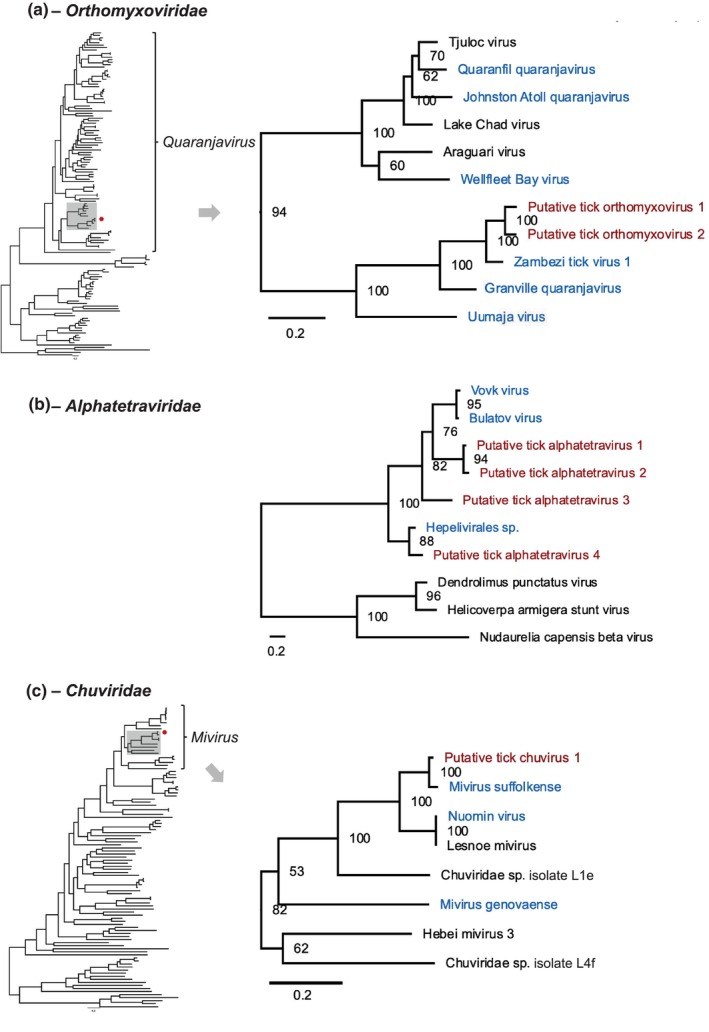
The maximum likelihood phylogeny of the putative novel viral contigs. Bootstrap values (the robustness of the nodes) are shown at the nodes, and the scale is given in amino acid substitutions per site. Putative novel viral contigs found in this study are shown in red, and previously known tick‐associated viruses are shown in blue. GenBank ID can be found at Figures A2, A3, A4 in Appendix 1. Alignments using ClustalW and maximum likelihood trees are provided in at https://github.com/ytlin2021/TickVirus.git.

**FIGURE A2:**
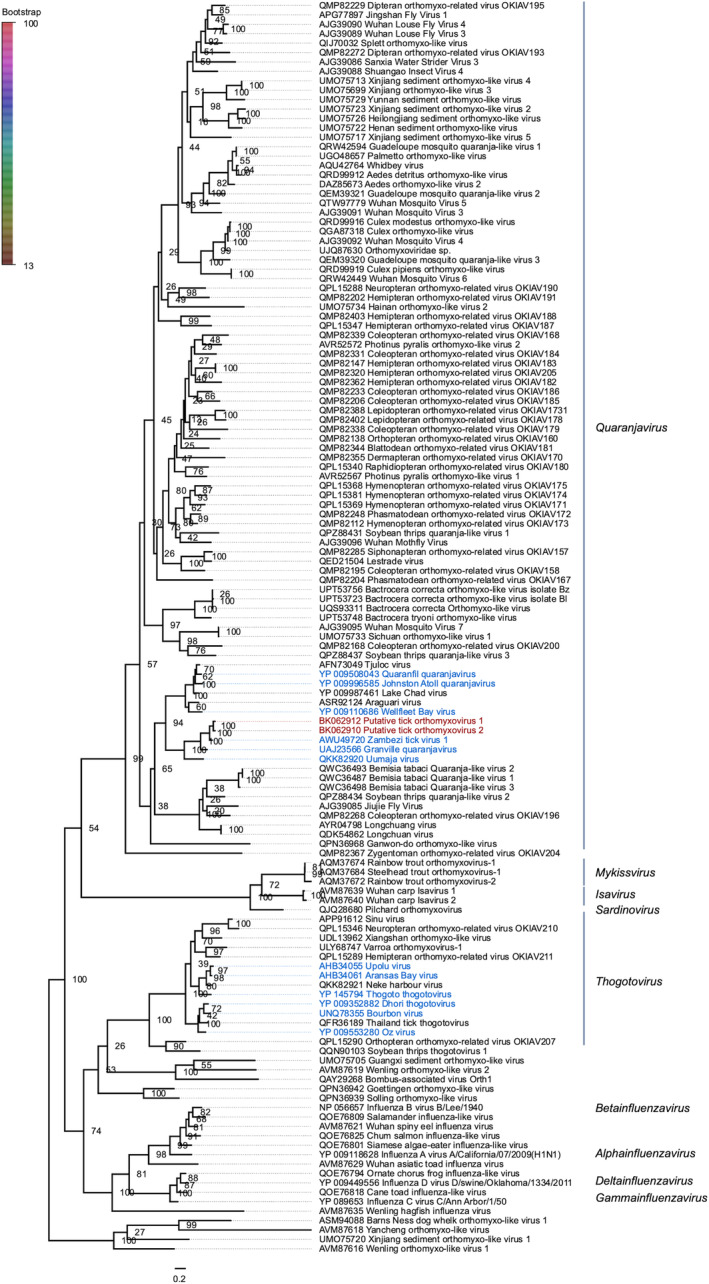
Phylogenetic relationship of the putative novel orthomyxoviruses. Maximum likelihood tree was inferred. Putative novel viral contigs found in this study are shown in red, and previously known tick‐associated viruses are shown in blue.

In this study, we discovered two putative novel tick orthomyxoviruses from ixodid tick metagenomic projects, one of which derived from sequence sampled from *Rhipicephalus sanguineus* in Italy and one from *Rhipicephalus haemaphysaloides* in China (Table 1). The viral contigs we found contained homologues of Influenza *PB1* gene segment, the most conserved of the orthomyxovirus genes (Figure 2a). Phylogenetic analysis revealed that the putative novel tick orthomyxoviruses fell into the genus *Quaranjavirus* and formed a separate clade with other tick‐associated quaranjaviruses (Figure 3a). Within PB1, the contigs were phylogenetically closely related to Zambezi tick virus 1—a highly divergent virus identified in *Rhipicephalus* ticks from Mozambique (Cholleti et al., 2018) and shared approximately 77% amino acids identity (Table 1). The complete maximum‐likelihood phylogeny of Orthomyxoviridae can be found in Figure A2 in Appendix 1.

#### 3.1.2 Putative novel tick alphatetraviruses

**FIGURE A3:**
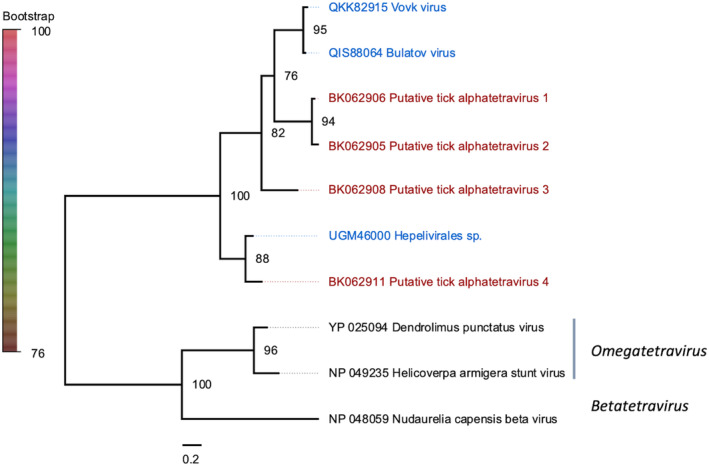
Phylogenetic relationship of the putative novel alphatetraviruses. Maximum likelihood tree was inferred. Putative novel viral contigs found in this study are shown in red, and previously known tick‐associated viruses are shown in blue.

The identified contigs in Alphatetraviridae ranged from 1488 to 5496 nt and comprised four putative viruses. In addition to an RdRp, some of the longer sequences also encoded putative nucleoside triphosphate hydrolase, viral RNA helicase and viral methyltransferase three motifs (Table 1; Figure 2b). Phylogenetic analysis suggested that the putative novel tick alphatetraviruses viruses formed a separate clade, being placed with the tick‐associated viruses recently identified in ixodid ticks from Antarctica (Vovk virus and Bulatov virus from the seabird tick, *Ixodes uriae*) (Wille et al., 2020) and China (Hubei tick hepe‐like virus found in *R. microplus* and *Haemaphysalis longicornis*) (Xu et al., 2021), and Heilongjiang sediment betatetravirus of unknown host, but originally sampled from river sediment in China (Chen et al., 2022) (Figure 3b). The complete maximum‐likelihood phylogeny of Alphatetraviridae can be found in Figure A3 in Appendix 1.

#### 3.1.3 Putative novel tick chuviruses

**FIGURE A4:**
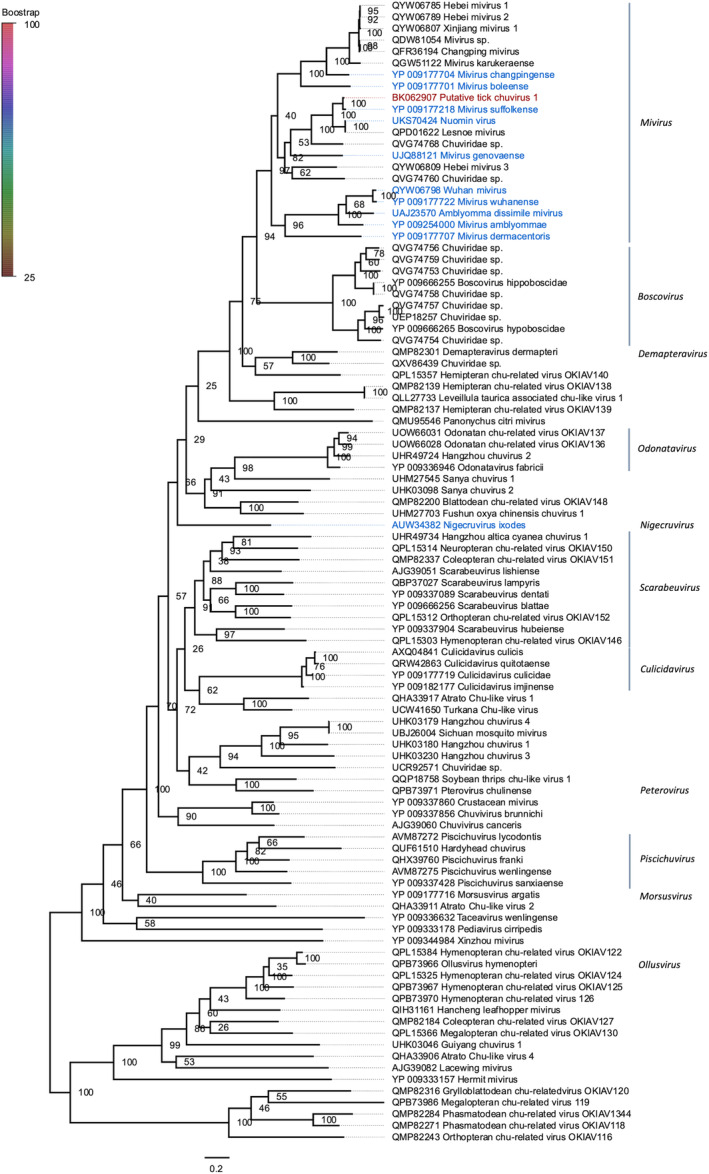
Phylogenetic relationship of the putative novel chuviruses. Maximum likelihood tree was inferred. Putative novel viral contigs found in this study are shown in red, and previously known tick‐associated viruses are shown in blue.

We identified 20 viral contigs in the Chuviridae from *Ixodes scapularis* and *Hyalomma asiaticum* sampled in the United States and China (Table 1; Table A3 in Appendix 1). Only the contig‐labelled Putative tick chuvirus 1 is consistent with a reproductively viable virus, which is 728 nt in length, comprising one putative ORF that encodes a 240‐amino acid protein containing the potential RdRp domain, nucleoside triphosphate hydrolase, viral RNA helicase and viral methyltransferase 3 (Table 1; Figure 2c). Phylogenetic analysis revealed that Putative tick chuvirus 1 was clustered with other miviruses and was closely related to Suffolk virus (*Mivirus suffolkense*), originally sampled in USA (Tokarz et al., 2018), with approximately 87% nucleotide identity (Figure 3c; Table 1). The complete maximum‐likelihood phylogeny of Chuviridae can be found in Figure A4 in Appendix 1. All other contigs showed signs of mutational degradation with many stop codons present in the region with homology to the RdRp (Table A3 in Appendix 1). All but one of the contigs exhibiting evidence of degradation were generated from a laboratory population of *Hy. asiaticum*, maintained under controlled conditions (Yuan et al., 2020). As such, we speculate that they likely result from the presence of an integrated chuvirus in the *Hy. asiaticum* genome present in this laboratory population, and, potentially, more broadly.

### 3.2 Zoonotic potential of tick‐associated viruses

#### 3.2.1 Assessment of known tick‐associated viruses with complete genomes

We used the prediction framework illustrated by Mollentze et al. (2021) to rank 133 known tick‐associated viruses with complete genomes; none were arthropod‐specific or microbiome infecting. The viruses here were ranked based on predicted mean zoonotic potential and further converted into four zoonotic potential categories, describing the overlap of confidence intervals (CIs) with the 0.293 cut‐off (see Section 2). The zoonotic prediction results are given in Table A6 in Appendix 1. This subset data set contained representatives from 13 viral families (29 genera) and unassigned viruses, including 21 viruses that were known to infect humans by our criteria.

**FIGURE 4:**
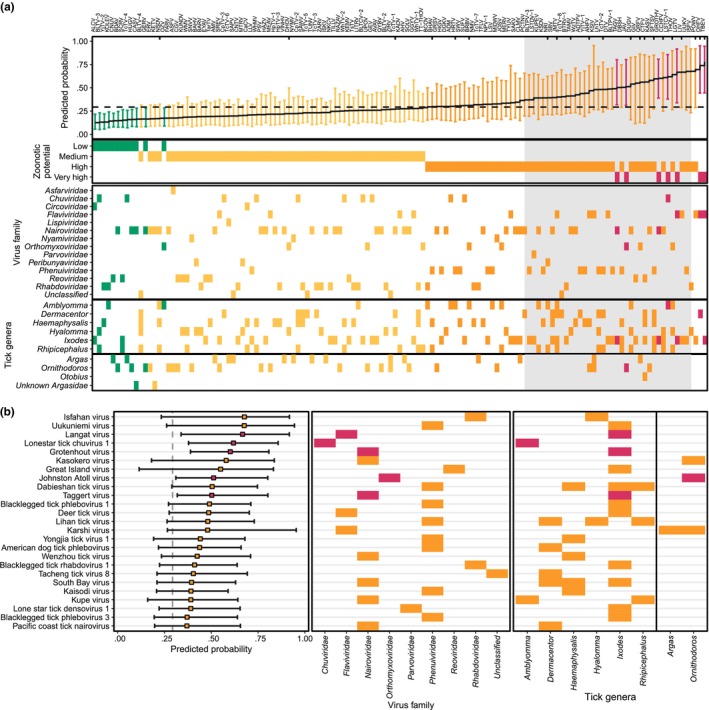
Predicted probability of human infection for tick viruses based on genome composition‐based model. (a) Predicted probability of human infection for 133 tick viruses with complete genomes. Colour scale show the assigned zoonotic potential categories (green: low; yellow: medium; orange: high; and pink: very high). Tick marks along the top edge of the first panel show the location of viruses known to infect humans, while a dashed line shows the cut‐off 0.293 that balanced sensitivity and specificity according to Mollentze et al. (2021). The top ranked 25 viruses that have no known human infection (contained within the grey box) are illustrated in more detail in (b). Points show the mean calibrated score, with lines indicating 95% confidence intervals. Figures were drawn in R v4.1.1. Virus names and zoonotic ranking results can be found in Table A6 in Appendix 1. Numeric data underlying this figure can be found at https://github.com/ytlin2021/TickVirus.git.

In total, 5.1% of tick‐associated viruses were identified as having very high zoonotic potential (*N* = 7), 39.7% having high zoonotic potential (*N* = 54), 44.1% having medium zoonotic potential (*N* = 60) and 11.0% having low zoonotic potential (*N* = 15; Figure A4 in Appendix 1). Among the 22 currently known human‐infecting tick‐associated viruses, 81.8% were correctly identified as having either very high (*N* = 2) or high zoonotic potential (*N* = 16), and the remaining human‐associated viruses were classified as medium zoonotic potential (*N* = 4; Figure A4 in Appendix 1). Among the tick‐associated viruses with unknown human infectivity that were sequenced from non‐human animal or tick samples, 37.7% were predicted to have either very high (*N* = 5) or high zoonotic potential (*N* = 38; Figure A4 in Appendix 1), including viruses in the family Chuviridae, Nyamiviridae and Parvoviridae that currently do not contain tick‐associated viruses known to infect humans. Langat virus, Lonestar tick chuvirus 1, Grotenhout virus, Taggert virus and Johnston Atoll virus were predicted to have very high zoonotic risk, although they were not known to infect humans (Figure 4b), suggesting a high priority for further virological research, in order to assess whether greater surveillance of them in wild tick populations would be warranted.

#### 3.2.2 Assessment of putative novel viruses with partial genomes

As the model was trained on complete genomes (Mollentze et al., 2021), it is inappropriate for the use on viruses for which only partial genomes are available. We therefore did qualitative assessment below by using the genomes of the closest related virus with a complete genome as a proxy and interpret with other clinical or epidemiological sources of evidence.

The six viral species in the same clade with the putative novel orthomyxoviruses had an average predicted risk of 0.367 (95% CI: 0.081–0.863) (Table 2). One viral species (*Johnston Atoll quaranjavirus*) was classified as very high zoonotic potential, and three viral species (*Wellfleet Bay virus*, *Tjuloc virus* and *Quaranfil quaranjavirus*) were categorised as having high zoonotic potential (Table 2; the GenBank ID can be found in Table A5 in Appendix 1), among which Quaranfil virus was known to infect humans (Mourya et al., 2019). The evidence indicates that the putative novel orthomyxoviruses identified in this study may represent a potential risk of human infection given exposure (which is possible, as *R. haemaphysaloides* is known to parasitise humans).

**TABLE 2:** Zoonotic risks of virus species closely related to the putative novel viruses.

	Viral species	Current status	Predicted probability	95% confidence interval	Zoonotic potential
Relative viral species of putative tick orthomyxoviruses	*Wellfleet Bay virus*	No known human infections	.368	0.213–0.636	High
	*Araguari virus*	No known human infections	.222	0.102–0.372	Medium
	*Johnston Atoll quaranjavirus*	No known human infections	.513	0.311–0.802	Very high
	*Lake Chad virus*	No known human infections	.184	0.081–0.334	Medium
	*Tjuloc virus*	No known human infections	.352	0.141–0.607	High
	*Quaranfil quaranjavirus*	Human virus	.561	0.128–0.863	High
Relative viral species of putative tick alphatetraviruses	*Nudaurelia capensis beta virus*	No known human infections	.302	0.153–0.497	High
	*Dendrolimus punctatus virus*	No known human infections	.234	0.128–0.352	Medium
	*Helicoverpa armigera stunt virus*	No known human infections	.243	0.135–0.387	Medium
Relative viral species of putative tick chuviruses	*Mivirus suffolkense*	No known human infections	.309	0.151–0.562	High

*Note*: Zoonotic risks of sequences in GenBank format were predicted using the genome composition‐based machine learning model, categorised into four priority categories (low: entire 95% CI of predicted probability ≤.293; medium: mean prediction ≤0.293, but CI crosses it; and high: mean prediction >0.293, but CI crosses it; very high: entire CI > 0.293) (25).

Putative tick chuvirus 1 showed a RdRp amino acid identity of around 90% with a recently described human infecting virus—Nuomin virus (Quan et al., 2020). Within the same clade, Suffolk virus was also categorised as having high zoonotic potential with a predicted probability of 0.309 (95% CI: 0.151–0.562) (Table 2; the GenBank IDs can be found in Table A5 in Appendix 1). Therefore, it is plausible that Putative tick chuvirus 1 would be assessed to have a high zoonotic risk. This may be especially relevant as the sequence was detected in *I. scapularis*, an important vector of other human pathogens, and so the potential for human exposure is high. However, given the small number of known species in the family Chuviridae when the GCB model was trained (Mollentze et al., 2021), caution must be applied, as the model might not be able to accurately predict the zoonotic potential viruses in this family.

Reported alphatetraviruses are almost exclusively arthropod‐associated (ICTV, 2022), with no alphatetravirus ever having been found infecting a mammal to the best of our knowledge. However, the model still predicted a medium or high zoonotic risk of the three published complete genomes of known alphatetraviruses with a mean predicted risk of 0.260 (95% CI: 0.128–0.497) (Table 2; the GenBank IDs can be found in Table A5 in Appendix 1). The disparity between the prediction outcomes and our existing knowledge of the viruses highlights either a deficiency of the currently available zoonotic risk assessment tools or our sampling of alphatetraviruses, discussed below.

## 4 DISCUSSION

Here, we reanalysed existing tick metagenomic and meta‐transcriptomic studies in public data sets to identify potential novel tick‐associated viruses, using a tick virus database containing all known tick‐associated viruses with genetic sequence available. We then employed the genome composition‐based machine learning model of Mollentze et al. (2021) to evaluate the zoonotic risk of all published tick viruses with complete genomes.

### 4.1 Novel virus discovery

The presence of undescribed viruses in pre‐existing meta‐genomic and ‐transcriptomic data illustrate a valuable underutilised data source. A combination of factors, ranging from studies being focused on specific taxa, limitations of the bioinformatic tools being used and incompleteness of reference datasets used within these tools, result in the generation of large amounts of unannotated or incorrectly annotated sequence. This provides an opportunity for small studies such as this one, or much larger studies considering wider ranges of or more extensively studied taxa (e.g. Käfer et al., 2019; Shi et al., 2016; Webster et al., 2015), reanalysing this historical data to discover new viruses (or, equally, other organisms of interest). As a pedagogical apart, as the resource requirements for these studies are low, the scope of the projects is limited, the outputs of general scientific interest, and the skills required of particular value to employers, these studies make excellent short projects for late‐stage undergraduate or early‐stage postgraduate students on ecology or ecology‐adjacent courses.

We have identified potential novel tick‐associated viruses belonging to three RNA viral families: Alphatetraviridae, Orthomyxoviridae and Chuviridae. Quaranjaviruses, which are enveloped negative‐sense single‐stranded segmented RNA viruses typically comprised of multiple segments (Mourya et al., 2019), have been previously documented in various hosts, including argasid and ixodid ticks, other arachnids, insects and vertebrates (Allison et al., 2015; Bratuleanu, Temmam, Chrétien, et al., 2022; Bratuleanu, Temmam, Munier, et al., 2022). In contrast, Alphatetraviridae members are non‐enveloped positive‐sense single‐stranded RNA viruses (ICTV, 2022). While these viruses were initially thought to primarily infect moths and butterflies (Erlandson, 2008), recent findings of alphatetraviruses in ticks (Wille et al., 2020; Xu et al., 2021) suggest that this perception may have been influenced by sampling biases. Supporting this notion, our study revealed the presence of potential alphatetraviruses in four ixodid tick species (*Ixodes ricinus*, *Ixodes persulcatus*, *Ornithodoros moubata* and *Rhipicephalus microplus*) sampled across three geographically diverse locations (China, Spain and USA) (Table 1). Chuviridae is a recently identified viral family of negative‐sense single‐stranded RNA viruses, containing representatives detected in both argasid and ixodid ticks (Li et al., 2015; Shi et al., 2016). Chuviruses occupy a unique position between segmented and unsegmented viruses in terms of phylogenetic diversity, displaying a wide variety of genome organisations, including unsegmented, bi‐segmented and circular forms (Li et al., 2015). Many newly discovered chuviruses have not been assigned to any viral genus (Figure A4 in Appendix 1). Li et al. (2015) proposed a novel group within the family; however, our study found that these putative unassigned chuviruses were placed on relatively distant branches of the phylogenetic tree (Figure 3), suggesting that virus classification within the Chuviridae family may be influenced by our limited understanding of its diversity. In this study, we identified only one DNA viral family, Asfarviridae, containing putative novel viruses. While the methods we used may be the cause of the paucity of DNA viruses we detected, this is consistent with the prior observation that this virome is primarily characterised by RNA viruses (Vandegrift & Kapoor, 2019).

As with all studies that base viral discovery around similarity to known viruses, our ability to discover novel viruses was limited by our reference data set. For instance, some newly discovered tick‐associated viruses (such as the viruses identified in Zhang et al., 2021 and Kong et al., 2022) were not included in the data set. Furthermore, a recent examination of tick metaviromes showed that approximately 70% of the 223 viruses identified in their study had never been previously documented in ticks (Ni et al., 2023). Hence, it is important to acknowledge that some putative novel tick‐associated viruses may have been missed due to the absence of similar viruses for comparison. Furthermore, our study underscores the importance of considering the ecological context when interpreting the presence of viruses in ticks. The mere detection of a virus within a tick does not necessarily indicate that the tick played a role in the transmission of that virus. It is instead possible that the tick acquired the virus while feeding on an infected vertebrate host, and the virus was present in the blood meal. Likewise, the detection of a virus infecting a tick does not imply that the tick's host is involved in the transmission of the virus. Understanding the dynamics of tick‐borne viral transmission requires a multifaceted approach. It involves not only identifying the viruses within ticks but also investigating the interplay between ticks, viruses and vertebrate hosts. This includes studying the genetic diversity, population structure and pathogen distribution in different tick species (e.g. Jia et al., 2020), the virus' ability to replicate within the tick (e.g. Mandl, 2005) and the mechanisms by which ticks may transmit the virus to new hosts (e.g. Bartíková et al., 2023; Jones & Nuttall, 1989; Kazimírová et al., 2017).

### 4.2 Zoonotic risk assessment

The genome composition‐based model applied showed promising results in predicting zoonotic risks of known tick‐associated viruses with complete genomes, particularly in distinguishing the zoonotic potential of closely related viruses within a genus. For instance, *Mivirus boleense* (Li et al., 2015) was ranked as low zoonotic potential, while *Mivirus suffolkense* (Tokarz et al., 2018) was deemed high zoonotic potential. Given the validation of the methodology previously performed, this adds to the evidence suggesting the use of such models in providing targets for further research in a quick and low‐cost way.

We noted that the high and very high zoonotic potential categories were dominated by Nairoviridae (31.0%) and Phenuiviridae (28.6%), consistent with the recent findings of emerging human‐infecting tick viruses in these two viral families. For example, among members of the Nairoviridae family, two emerging orthonairoviruses called Songling virus and Yezo virus have been recently discovered in *Hyalomma* spp. ticks and isolated in patients with the acute febrile disease (Kodama et al., 2021; Ma et al., 2021). Hospitals in Inner Mongolia and Heilongjiang province, China also reported infections from a novel nairovirus named Beiji nairovirus which mainly circulates in the ixodid ticks *I. crenulatus* and *I. persulcatus* (Wang et al., 2021). Similarly, among the Phenuiviridae, a novel phlebovirus named Tacheng tick virus 2 was first sampled from *D. marginatus* ticks in China (Li et al., 2015) and later identified in various countries and tick species, primarily circulating in *D. marginatus* and *H. marginatum* ticks (Bratuleanu, Temmam, Chrétien, et al., 2022; Bratuleanu, Temmam, Munier, et al., 2022; Brinkmann et al., 2018; Temmam et al., 2019). The risk for human infection from Tacheng tick virus 2 was not known until 2021 when Dong et al. (2021) reported on Tacheng tick virus 2 infection in a patient in China. The wide distribution of these tick species suggests that the geographic limits of the emerging tick‐associated viruses in the family Nairoviridae and Phenuiviridae may be larger than presently assumed.

A significant proportion of tick‐associated viruses with high or very high zoonotic potential were sampled from ixodid ticks, which may reflect sampling bias towards viruses infecting hard ticks (family Ixodidae) in previous studies or be true representation of differences in intrinsic zoonotic risk between the viral communities of argasid and ixodid ticks. The ecology of argasid and ixodid ticks are different, with ixodid ticks generally having longer blood meals (Lawrie et al., 2004). The longer feeding time of ixodid ticks may make them, on average, more favourable for viral transmission and thus more likely to harbour communities of viruses capable of infecting mammals. However, it also means that ixodid ticks are more likely to be detected on a host, and thus more likely to be associated with resulting viral infections (i.e. a virus that might cause a fever of unknown origin if transmitted by an argasid tick may be able to be explicitly linked to a tick bite given the greater discovery probability if transmitted by an ixodid tick). A more systematic approach that samples both hard and soft ticks would increase our knowledge of the diversity of tick‐associated viruses carried by each type and allow us to assess whether they truly do differ in human infection risk.

Caution must be taken when interpreting the results from the zoonotic risk assessment model, First, the predicted probability intervals were often large (as demonstrated in Figure 4b), leading to low confidence in the actual zoonotic risk of these viruses, and some viruses' zoonotic risks were inaccurately estimated. For example, Issyk‐Kul virus had low predicted probability and associated ‘medium’ risk, although it has been indicated as a likely human pathogen (L'vov et al., 1984). More importantly, these tools face challenges when attempting to extrapolate beyond the training data set's distribution. One potential example of this is the results observed in the Alphatetraviridae, discussed above. No alphatetravirus has ever been identified in a mammal; yet, several were predicted to have medium or high zoonotic potential. It is unclear whether this represents a systematic error with the models' predictions, or an indication that there are clades within Alphatetraviridae that may be capable of the infection of vertebrates. The presence of alphatetraviruses in ticks of Antarctic penguins (Wille et al., 2020) could potentially suggest an avenue to investigate this question. Dense sampling of the sympatric penguins could be used to search for corresponding active infections in the hosts or serological evidence of past infection. Irrespective of whether these specific alphatetravirus assessments are in error, expanding the diversity of the training data to zoonotic prediction models is likely to increase predictive accuracy. Notwithstanding the issues above, good performance was observed in the family Nairoviridae, which included only 13 viruses in the data set with the model predicting high risks of zoonotic potential for viruses with the group, consistent with recent findings of new and emerging tick‐associated viral diseases associated with nairoviruses and orthonairoviruses (Kodama et al., 2021; Ma et al., 2021; Moming et al., 2021; Wang et al., 2021).

Another limitation of the GCB model is its training on complete genomes (Mollentze et al., 2021), which restricts the application of the model on viruses for which only partial genomes are available. This is a particularly important limitation for viruses found purely through bioinformatic means, where only single contigs or disconnected viral segments may be available, which impacts the ability to make fast decisions on virus research and surveillance at the earliest stage of virus discovery. We attempted to assess the zoonotic risk of the putative novel viruses described in this study using the genomes of the closest related virus with a complete genome as a proxy. However, while this was unavoidable in this case, it does fall prey to one of the same biases the use of genome‐based composition methods was originally intended to avoid, the assumption that zoonotic risk is conserved across the phylogeny. Hence, the zoonotic risks of these viruses ought to be reassessed should full genomes become available. Training existing models on features from partial viral genomes also provides a potential avenue for future work to help alleviate this issue.

## 5 CONCLUSION

Despite ticks being important vectors of pathogens, the human infection risks of most tick‐associated viruses remain uncertain (Shi et al., 2018). In this study, we have both identified new tick viruses in previously collected data and assessed the zoonotic potential of known tick virus diversity, identifying certain high risk viral families deserving of further study and, potentially, surveillance. Cataloguing viruses and assessing their human infection risk, as we have done here, represents only a first step in the risk management of zoonoses. Further research into the ecological processes that underlie the geographical distribution and interspecific transmission of ticks and their viruses is necessary to gain a comprehensive understanding of the ecology of this system. It is this eventual ecological knowledge that may allow us to formulate more effective strategies for managing human exposure to vectors, preventing zoonoses at the source.

## AUTHOR CONTRIBUTIONS

**Yuting Lin:** Data curation (lead); formal analysis (equal); investigation (lead); methodology (equal); project administration (lead); visualization (lead); writing – original draft (lead); writing – review and editing (equal). **David J. Pascall:** Conceptualization (lead); formal analysis (equal); funding acquisition (lead); methodology (equal); resources (lead); supervision (lead); writing – review and editing (equal).

## CONFLICT OF INTEREST STATEMENT

The authors have no conflict of interest to declare.

## Data Availability

All novel viral sequences identified in this study have been submitted to Third Party Annotation Section of the DDBJ/ENA/GenBank databases under the accession numbers TPA: BK062905–BK062908, BK062910–BK062912. Data and scripts used to generate the analyses are provided in https://github.com/ytlin2021/TickVirus.git.

## APPENDIX 1

**TABLE A1:** Summary of tick‐associated viruses by December 2021, used for tick virus genetic database building. Information from a wide variety of sources, references in the table.

Virus	Species	Genus	Family	Genome structure	Accession	Available sequence	Human infection	Earliest accessible report	Tick vectors, likely incomplete	Distribution
African swine fever virus	*African swine fever virus*	*Asfivirus*	Asfarviridae	dsDNA	NC_044942	Complete genome	?	1921 (Eustace Montgomery, 1921)	*O. erraticus*, *O. moubata* (Shi et al., 2018)	Sub‐Saharan Africa, South America, Southern Europe (Shi et al., 2018)
Canine circovirus	*Canine circovirus*	*Circovirus*	Circoviridae	ssDNA (+/−)	NC_020904	Complete genome	?	2012 (Kapoor et al., 2012)	*Haemaphysalis* spp., *Rhipicephalus* spp. (Wang, Wang, et al., 2020; Wang, Zhao, et al., 2020)	Australia, Brazil, China, Columbia, Croatia, Germany, Iran, Italy, Norway, Thailand, Turkey, UK, USA, Viet Nam (Urbani et al., 2021)
Avian‐like circovirus	*Tick associated circovirus 1*	*Circovirus*	Circoviridae	ssDNA (+/−)	NC_030199	Complete genome	?	2018 (Tokarz et al., 2018)	*I. scapularis* (Tokarz et al., 2018)	USA (Tokarz et al., 2018)
Nayun tick torquevirus	Unclassified	*Thetatorquevirus*	Anelloviridae	ssDNA (+/−)	KP141758	Partial genome	?	2015 (Xia et al., 2015)	*Rhipicephalus* spp. (Xia et al., 2015)	China (Xia et al., 2015)
Lone star tick densovirus 1	Unclassified	Unclassified	Parvoviridae	ssDNA (+/−)	KX774632	Complete genome	?	2018 (Tokarz et al., 2018)	*I. scapularis* (Tokarz et al., 2018)	USA (Tokarz et al., 2018)
Colorado tick fever virus	*Colorado tick fever coltivirus*	*Coltivirus*	Reoviridae	dsRNA	NC_004191, NC_004181, NC_004182, NC_004183, NC_004184, NC_004185, NC_004186, NC_004187, NC_004188, NC_004180, NC_004189, NC_004190	Complete genome	Y (Shi et al., 2018)	1946 (Florio & Stewart, 1947)	*D. andersoni*, *D. albipictus*, *D. arumapertus*, D. occidentalis, *H. leporispalustris*, *I. sculptus*, I. spinipalpis, *Ot. lagophilus* (Shi et al., 2018)	USA (Shi et al., 2018)
Eyach virus	*Eyach coltivirus*	*Coltivirus*	Reoviridae	dsRNA	NC_003696, NC_003697, NC_003698, NC_003699, NC_003700, NC_003701, NC_003702, NC_003703, NC_003704, NC_003705, NC_003706, NC_003707	Complete genome	?	1976 (Rehse‐Küpper et al., 1976)	*I. ricinus*, *I. ventalloi* (Shi et al., 2018)	France, Germany (Shi et al., 2018)
Kundal virus	*Kundal coltivirus*	*Coltivirus*	Reoviridae	dsRNA	NC_055248, NC_055249, NC_055247, NC_055240, NC_055250, NC_055239, NC_055242, NC_055245, NC_055241, NC_055246, NC_055243, NC_055244	Complete genome	?	2019 (Yadav et al., 2019)	*Hy. antolicum* (Yadav et al., 2019)	India (Yadav et al., 2019)
Tarumizu tick virus	*Tarumizu coltivirus*	*Coltivirus*	Reoviridae	dsRNA	NC_055255, NC_055254, NC_055261, NC_055259, NC_055262, NC_055251, NC_055260, NC_055258, NC_055252, NC_055257, NC_055253, NC_055256	Complete genome	?	2017 (Fujita et al., 2017)	*H. flava* (Fujita et al., 2017)	Japan (Fujita et al., 2017)
Chenuda virus	*Chenuda virus*	*Orbivirus*	Reoviridae	dsRNA	NC_027534, NC_027535, NC_027536, NC_027537, NC_027538, NC_027548, NC_027549, NC_027550, NC_027551, NC_027552	Complete genome	?	1978 (Karabatsos, 1978)	*A. hermanni* (Shi et al., 2018)	Egypt, Uzbekistan (Shi et al., 2018)
Chobar Gorge virus	*Chobar Gorge virus*	*Orbivirus*	Reoviridae	dsRNA	NC_027553, NC_027554, NC_027555, NC_027556, NC_027557, NC_027558, NC_027559, NC_027560, NC_027561, NC_027562	Complete genome	?	1978 (Karabatsos, 1978)	*Ornithodoros* spp. (Shi et al., 2018)	Nepal (Shi et al., 2018)
Great Island virus	*Great Island virus*	*Orbivirus*	Reoviridae	dsRNA	NC_014522, NC_014523, NC_014524, NC_014525, NC_014526, NC_014527, NC_014528, NC_014529, NC_014530, NC_014531	Complete genome	?	1981 (Nuttall et al., 1981)	*I. uriae* (Shi et al., 2018)	Canada (Shi et al., 2018)
Kemerovo virus	*Great Island virus*	*Orbivirus*	Reoviridae	dsRNA	MF939555, MF939556, MF939557, MF939558, MF939559, MF939560, MF939561, MF939562, MF939563, MF939564, KJ010787	Complete genome	?	1970 (Libíková et al., 1970)	*I. persulcatus*, *I. ricinus* (Shi et al., 2018)	Russia, Slovakia (Shi et al., 2018)
Lipovnik virus	*Great Island virus*	*Orbivirus*	Reoviridae	dsRNA	HM543475, HM543476, HM543477	Partial genome	?	1964 (Libíková et al., 1964)	*I. ricinus* (Shi et al., 2018)	Czech Republic, Slovakia (Shi et al., 2018)
Tribec virus	*Great Island virus*	*Orbivirus*	Reoviridae	dsRNA	HQ266581, HQ266582, HQ266583, HQ266584, HQ266585, HQ266586, HQ266587, HQ266588, HQ266589, HQ266590	Partial genome	?	1964 (Libíková et al., 1964)	*H. punctata*, *I. ricinus* (Shi et al., 2018)	Belorussia, Italy, Slovakia (Shi et al., 2018)
St Croix River virus	*St Croix River virus*	*Orbivirus*	Reoviridae	dsRNA	NC_006006, NC_005998, NC_005999, NC_006000, NC_006001, NC_006002, NC_006003.1, NC_006004, NC_006005, NC_005997	Complete genome	?	2001 (Attoui et al., 2001)	*I. scapularis*, *R. appendiculatus* (Shi et al., 2018)	Kenya, USA (Alberdi et al., 2012)
Baku virus	Unclassified	*Orbivirus*	Reoviridae	dsRNA	KY023349, KY023350, KY023351, KY023352, KY023353, KY023354, KY023355, KY023356, KY023357, KY023358	Partial genome	?	1978 (Karabatsos, 1978)	*O. maritimus* (Shi et al., 2018)	Caspian Sea, Uzbekistan (Shi et al., 2018)
Matucare virus	Unclassified	*Orbivirus*	Reoviridae	dsRNA	HQ397640	Partial genome	?	1970 (Justines & Kuns, 1970)	*Ot. megnini* (Palacios et al., 2011)	
Okhotskiy virus	Unclassified	*Orbivirus*	Reoviridae	dsRNA	KY023329, KY023330, KY023331, KY023332, KY023333, KY023334, KY023335, KY023336, KY023337, KY023338, KF981623, KF981624, KF981625, KF981626, KF981627, KF981628, KF981629, KF981630, KF981631, KF981632, MN830241, MN830242, MN830243, MN830244, MN830245	Partial genome	?	1878 (L'vov, Timopheeva, et al., 1973)	*I. uriae* (Pettersson et al., 2020)	Northern Sweden, Okhotsk (Pettersson et al., 2020)
Wad Medani virus	*Wad Medani virus*	*Orbivirus*	Reoviridae	dsRNA	NC_027533, NC_027539, NC_027540, NC_027541, NC_027542, NC_027543, NC_027544, NC_027545, NC_027546, NC_027547	Complete genome	?	1966 (Taylor et al., 1966)	*Hyalomma* spp., *R. sanguineus* (Shi et al., 2018)	Asia, East Africa, Jamaica (Shi et al., 2018)
Norway partiti‐like virus 1	Unclassified	Unclassified	Partitiviridae	dsRNA	MF141076	Partial genome	?	2017 (Pettersson et al., 2017)	*I. ricinus* (Pettersson et al., 2017)	Norway (Pettersson et al., 2017)
Lonestar tick totivirus	Unclassified	Unclassified	Totiviridae	dsRNA	MG647775, MZ567079	Partial genome	?	2018 (Tokarz et al., 2018)	*I. scapularis* (Tokarz et al., 2018)	USA (Tokarz et al., 2018)
Fennesvirus	Unclassified	Unclassified	Unclassified Riboviria	dsRNA	MT025166, MT025167, MT025168, MT025169, MN830240	Partial genome	?	2020 (Wille et al., 2020)	*I. uriae* (Wille et al., 2020)	Antarctica (Wille et al., 2020)
Shelly Beach virus	Unclassified	Unclassified	Unclassified Riboviria	dsRNA	MK026600, MK026601, MK026602, MK026603, MK026604, MK026605	Partial genome	?	2018 (Harvey et al., 2019)	*I. holocyclus* (Harvey et al., 2019)	Australia (Harvey et al., 2019)
Shelly headland virus	Unclassified	Unclassified	Unclassified Riboviria	dsRNA	OL452144, OL452150, OL452158, OL452171, OL452185, OL452194, OL452203, OL452214, OL452222, OL452151, OL452172, OL452215, OL452223, OL452173, OL452216, OL452224, OL452174, OL452217, OL452225, OL452175, OL452226, OL452218, OL452227, OL452228, OL452176, OL452178, OL452229, OL452179, OL452219, OL452230, OL452180, OL452220, OL452231, OL452232, OL452181, OL452221, OL452233, OL452182, OL452234, OL452235, MK026577, MK026581, MK026582	Partial genome	?	2018 (Harvey et al., 2019)	*I. holocyclus* (Harvey et al., 2019)	Australia (Harvey et al., 2019)
Lonestar tick chuvirus 1	*Mivirus amblyommae*	*Mivirus*	Chuviridae	ssRNA(−)	NC_030204	Complete genome	?	2018 (Tokarz et al., 2018)	*Am. americanum* (Tokarz et al., 2018)	USA (Tokarz et al., 2018)
Bole tick virus 3	*Mivirus boleense*	*Mivirus*	Chuviridae	ssRNA(−)	NC_028259	Complete genome	?	2015 (Li et al., 2015)	*Hy. asiaticum*, *Hy. aegyptium*, *R. turanicus*, *R. bursa* (Brinkmann et al., 2018; Ergünay et al., 2020; Li et al., 2015)	China, Turkey (Brinkmann et al., 2018; Ergünay et al., 2020; Li et al., 2015)
Changping tick virus 2	*Mivirus changpingense*	*Mivirus*	Chuviridae	ssRNA(−)	NC_028260	Complete genome	?	2015 (Li et al., 2015)	*Dermacentor* spp., *H. parva*, *R. sanguineus* (Li et al., 2015; Temmam et al., 2019)	China, Thailand, Turkey, Trinidad, Tobago (Brinkmann et al., 2018; Li et al., 2015; Temmam et al., 2019)
Changping tick virus 3	*Mivirus dermacentoris*	*Mivirus*	Chuviridae	ssRNA(−)	NC_028261	Complete genome	?	2015 (Li et al., 2015)	*Dermacentor* spp. (Li et al., 2015)	China (Li et al., 2015)
Genoa virus	*Mivirus genovaense*	*Mivirus*	Chuviridae	ssRNA(−)	OL452069	Complete genome	?	2018 (Harvey et al., 2019)	*I. holocyclus* (Harvey et al., 2019)	Australia (Harvey et al., 2019)
Suffolk virus	*Mivirus suffolkense*	*Mivirus*	Chuviridae	ssRNA(−)	NC_028243	Complete genome	?	2018 (Tokarz et al., 2018)	*I. scapularis* (Tokarz et al., 2018)	USA (Tokarz et al., 2018)
Wuhan tick virus 2	*Mivirus wuhanense*	*Mivirus*	Chuviridae	ssRNA(−)	NC_028266	Complete genome	?	2015 (Li et al., 2015)	*H. longicornis*, *R. microplus* (Gómez et al., 2020; Xu et al., 2021)	Brazil, China, Colombia, Guadeloupe, Thailand, Trinidad, Tobago (Gómez et al., 2020; Xu et al., 2021)
Wuhan mivirus	*Wuhan mivirus*	*Mivirus*	Chuviridae	ssRNA(−)	MZ244267	Complete genome	?	2015 (Li et al., 2015)	*H. longicornis*, *R. microplus*, *R. sanguineus* (Sameroff et al., 2019)	China, Thailand, Trinidad and Tobago (Sameroff et al., 2019)
Tacheng tick virus 4	*Morsusvirus argatis*	*Morsusvirus*	Chuviridae	ssRNA(−)	NC_028263	Complete genome	?	2015 (Li et al., 2015)	*A. miniatus* (Li et al., 2015)	China (Li et al., 2015)
Blacklegged tick chuvirus 2	*Nigecruvirus ixodes*	*Nigecruvirus*	Chuviridae	ssRNA(−)	MF360789	Complete genome	?	2018 (Tokarz et al., 2018)	*I. scapularis* (Tokarz et al., 2018)	USA (Tokarz et al., 2018)
Amblyomma dissimile mivirus^a^	Unclassified	*Mivirus*	Chuviridae	ssRNA(−)	MZ502309.1	Complete genome	?	2021 (Sameroff et al., 2021)	*Am. dissimile* (Sameroff et al., 2021)	Trinidad and Tobago (Sameroff et al., 2021)
Nuomin virus^a^	Unclassified	Unclassified	Chuviridae	ssRNA(−)	MW029986.1	Partial genome	Y (Quan et al., 2020)	2020 (Quan et al., 2020)	*I. persulcatus*, *I. crenulatus*, *H. conicinna*, *H. longicornis* (Quan et al., 2020)	China (Quan et al., 2020)
Tacheng tick virus 6	*Tacheng arlivirus*	*Arlivirus*	Lispiviridae	ssRNA(−)	NC_031270	Complete genome	?	2015 (Li et al., 2015)	*A. miniatus* (Li et al., 2015)	China (Li et al., 2015)
Grotenhout virus	*Grotenhout norwavirus*	*Norwavirus*	Nairoviridae	ssRNA(−)	KY700684, KY700683	Complete genome	?	2017 (Vanmechelen et al., 2017)	*I. ricinus* (Vanmechelen et al., 2017)	Belgium (Vanmechelen et al., 2017)
Abu Hammad virus	*Abu Hammad orthonairovirus*	*Orthonairovirus*	Nairoviridae	ssRNA(−)	KU925434, KU925435, KU925436	Complete genome	?	1976 (Darwish et al., 1976)	*A. hermanni* (Darwish et al., 1976)	Egypt (Darwish et al., 1976)
Abu Mina virus	*Abu Mina orthonairovirus*	*Orthonairovirus*	Nairoviridae	ssRNA(−)	KU925437, KU925438, KU925439	Complete genome	?	1976 (Darwish et al., 1976)	*A. streptopelia* (Darwish et al., 1976)	Egypt (Darwish et al., 1976)
Artashat virus	*Artashat orthonairovirus*	*Orthonairovirus*	Nairoviridae	ssRNA(−)	NC_043440, NC_043442, NC_043441	Partial genome	?	2014 (Al'khovskiĭ et al., 2014)	*O. alactagalis* (Al'khovskiĭ et al., 2014)	Armenia, Azerbaijan (Al'khovskiĭ et al., 2014)
Avalon virus	*Avalon orthonairovirus*	*Orthonairovirus*	Nairoviridae	ssRNA(−)	NC_040460, NC_040459, NC_040458	Complete genome	?	1976 (Main et al., 1976)	*I. uriae* (Pettersson et al., 2020)	Canada, France, Sweden (Pettersson et al., 2020)
Burana virus	*Burana orthonairovirus*	*Orthonairovirus*	Nairoviridae	ssRNA(−)	NC_043439, NC_043438, NC_043437	Partial genome	?	2014 (L'vov et al., 2014)	*H. punctate* (L'vov et al., 2014)	Kyrgyzstan (L'vov et al., 2014)
Chim virus	*Chim orthonairovirus*	*Orthonairovirus*	Nairoviridae	ssRNA(−)	NC_043434, NC_043436, NC_043435	Partial genome	?	2014 (L'vov et al., 2014)	*O. tartakovskyi* (L'vov et al., 2014)	Uzbekistan (L'vov et al., 2014)
Crimean‐Congo hemorrhagic fever virus	*Crimean‐Congo hemorrhagic fever orthonairovirus*	*Orthonairovirus*	Nairoviridae	ssRNA(−)	NC_005301, NC_005300, NC_005302	Complete genome	Y (Garrison et al., 2020)	1945 (Grashchenkov, 1944)	*Hy. asiaticum*, *Hy. marginatum*, *Ixodae* spp., *R. rossicus* (Shi et al., 2018; Zhao et al., 2021)	Many countries in Asia and Africa; parts of Europe (e.g., Albania, Bulgaria) (Shi et al., 2018)
Dera Ghazi Khan virus	*Dera Ghazi Khan orthonairovirus*	*Orthonairovirus*	Nairoviridae	ssRNA(−)	NC_034520, NC_034510, NC_034521	Complete genome	?	1970 (Begum et al., 1970)	*Hy. dromedarii* (Begum et al., 1970)	Pakistan (Begum et al., 1970)
Dugbe virus	*Dugbe orthonairovirus*	*Orthonairovirus*	Nairoviridae	ssRNA(−)	NC_004159, NC_004158, NC_004157	Complete genome	Y (Garrison et al., 2020)	1964 (Causey, 1970)	*Am. variegatum*, *B. decoloratus*, *Hy. truncatum*, and *R. appendiculatus* (Shi et al., 2018)	Sub‐Saharan Africa (Shi et al., 2018)
Estero Real virus	*Estero Real orthonairovirus*	*Orthonairovirus*	Nairoviridae	ssRNA(−)	MH017280, MH017286, MH017274	Complete genome	?	1985 (Málková et al., 1985)	*O. tadaridae* (Málková et al., 1985 *)*	Cuba (Málková et al., 1985)
Hazara virus	*Hazara orthonairovirus*	*Orthonairovirus*	Nairoviridae	ssRNA(−)	NC_038709, NC_038710, NC_038711	Complete genome	?	1970 (Begum et al., 1970)	*I. redikorzevi* (Begum et al., 1970)	Japan, Pakistan (Begum et al., 1970; Matsumoto et al., 2019)
Huangpi tick virus 1	*Huangpi orthonairovirus*	*Orthonairovirus*	Nairoviridae	ssRNA(−)	NC_031135, NC_031136, NC_031137	Complete genome	?	2015 (Li et al., 2015)	*H. doenitzi*, *H. longicornis* (Shi et al., 2018)	China (Shi et al., 2018)
Caspiy virus	*Hughes orthonairovirus*	*Orthonairovirus*	Nairoviridae	ssRNA(−)	KP792708, KP792709, KP792710	Complete genome	?	2014 (L'vov et al., 2014)	Various *Argasidae* (L'vov et al., 2014)	Azerbaijan (L'vov et al., 2014)
Farallon virus	*Hughes orthonairovirus*	*Orthonairovirus*	Nairoviridae	ssRNA(−)	NC_034502, NC_034494, NC_034503	Complete genome	?	1967 (Rodovsky & Stiller, 1967)	*Ornithodorus* spp. (Shi et al., 2018)	USA (Shi et al., 2018)
Great Saltee virus	*Hughes orthonairovirus*	*Orthonairovirus*	Nairoviridae	ssRNA(−)	KU925467, KU925468, KU925469	Complete genome	?	1976 (Keirans et al., 1976)	*O. maritimus* (Kuhn et al., 2016)	Ireland (Great Saltee Island) (Kuhn et al., 2016)
Hughes virus	*Hughes orthonairovirus*	*Orthonairovirus*	Nairoviridae	ssRNA(−)	KU925470, KU925471, KU925472	Complete genome	?	1964 (Hughes et al., 1964)	*O. denmarki* (Shi et al., 2018)	Cuba, Trinidad, USA, Venezuela (Shi et al., 2018)
Raza virus	*Hughes orthonairovirus*	*Orthonairovirus*	Nairoviridae	ssRNA(−)	KU925479, KU925480, KU925481	Complete genome	?	1968 (Clifford et al., 1969)	*O. denmarki* (Kuhn et al., 2016)	Mexico (Kuhn et al., 2016)
Issyk‐kul virus	*Issyk‐kul orthonairovirus*	*Orthonairovirus*	Nairoviridae	ssRNA(−)	KF892055, KF892056, KF892057	Complete genome	Y (L'vov et al., 1984)	1973 (L'vov, Karas, et al., 1973)	*A. vespertlionis* (L'vov, Karas, et al., 1973)	Kyrgyzstan (Atkinson et al., 2015)
Kasokero virus	*Kasokero orthonairovirus*	*Orthonairovirus*	Nairoviridae	ssRNA(−)	NC_036636, NC_029933, NC_029932	Complete genome	?	1986 (Kalunda et al., 1986)	*O. faini* (Schuh et al., 2020)	Uganda (Walker et al., 2015)
Keterah virus	*Keterah orthonairovirus*	*Orthonairovirus*	Nairoviridae	ssRNA(−)	NC_034392, NC_034388, NC_034389	Complete genome	?	1976 (Varma & Converse, 1976)	*Argasidae* (Varma & Converse, 1976)	Malaysia (Varma & Converse, 1976)
Kupe virus	*Kupe orthonairovirus*	*Orthonairovirus*	Nairoviridae	ssRNA(−)	EU257628, EU257627, EU257626	Complete genome	?	2009 (Crabtree et al., 2009)	*Am. gemma*, *R. pulchellus* (Crabtree et al., 2009)	Kenya (Crabtree et al., 2009)
Meram virus	*Meram orthonairovirus*	*Orthonairovirus*	Nairoviridae	ssRNA(−)	MN972594, MN972595, MN972596	Complete genome	?	2020 (Ergünay et al., 2020)	*Hy. aegyptium* (Ergünay et al., 2020)	Turkey (Ergünay et al., 2020)
Nairobi sheep disease virus	*Nairobi sheep disease orthonairovirus*	*Orthonairovirus*	Nairoviridae	ssRNA(−)	NC_034387, NC_034391, NC_034386	Complete genome	Y (Yadav et al., 2011)	1969 (Dandawate & Shah, 1969)	*Am. variegatum*, *H. longicornis*, *H. intermedia*, *R. appendiculatus*, *R. hemaphysaloides* (Krasteva et al., 2020)	China, Kenya, India (Krasteva et al., 2020)
Pacific coast tick nairovirus	*Pacific Coast orthonairovirus*	*Orthonairovirus*	Nairoviridae	ssRNA(−)	KU933933, KU933934, KU933935	Complete genome	?	2017 (Bouquet et al., 2017)	*D. occidentalis* (Bouquet et al., 2017)	USA (Bouquet et al., 2017)
Punta Salinas virus	*Punta orthonairovirus*	*Orthonairovirus*	Nairoviridae	ssRNA(−)	KU925473, KU925474, KU925475	Complete genome	?	1981 (Converse et al., 1981)	*O. amblus* (Kuhn et al., 2016)	Peru (Kuhn et al., 2016)
Bandia virus	*Qalyub orthonairovirus*	*Orthonairovirus*	Nairoviridae	ssRNA(−)	KU925446, KU925447, KU925448	Complete genome	?	1967 (Brès et al., 1967)	*O. sonrai* (Kuhn et al., 2016)	Senegal (Kuhn et al., 2016)
Geran virus	*Qalyub orthonairovirus*	*Orthonairovirus*	Nairoviridae	ssRNA(−)	KP792714, KP792715, KP792716	Complete genome	?	2014 (L'vov et al., 2014)	*O. asperus* (Kuhn et al., 2016)	Azerbaijan (Kuhn et al., 2016)
Qalyub virus	*Qalyub orthonairovirus*	*Orthonairovirus*	Nairoviridae	ssRNA(−)	NC_034511, NC_034522, NC_034512	Complete genome	?	1952 (Kuhn et al., 2016)	*O. asperus*, *O. erraticus* (Kuhn et al., 2016; Walker et al., 2016)	Azerbaijan, Egypt, Senegal (Kuhn et al., 2016; Walker et al., 2016)
Clo Mor virus	*Sakhalin orthonairovirus*	*Orthonairovirus*	Nairoviridae	ssRNA(−)	NC_034561, NC_034554, NC_034562	Complete genome	?	1976 (Main et al., 1976)	*A. cooleyi*, *I. uriae* (Walker et al., 2016)	UK, USA (Walker et al., 2016)
Sakhalin virus	*Sakhalin orthonairovirus*	*Orthonairovirus*	Nairoviridae	ssRNA(−)	KU925482, KU925483, KU925484	Complete genome	?	1972 (L'vov et al., 1972)	*I. uriae* (Kuhn et al., 2016)	Antarctica, Australia, Russia, USA (Kuhn et al., 2016; L'vov et al., 2014; Pettersson et al., 2020)
Taggert virus	*Sakhalin orthonairovirus*	*Orthonairovirus*	Nairoviridae	ssRNA(−)	KU925491, KU925492, KU925493	Complete genome	?	1985 (St George et al., 1985)	*I. uriae* (Pettersson et al., 2020; Wille et al., 2020)	Antarctica, Australia (Pettersson et al., 2020; Wille et al., 2020)
Tillamook virus	*Sakhalin orthonairovirus*	*Orthonairovirus*	Nairoviridae	ssRNA(−)	KU925494, KU925495, KU925496	Complete genome	?	1973 (Thomas et al., 1973)	*I. uriae* (Kuhn et al., 2016)	USA (Kuhn et al., 2016)
Soldado virus	*Soldado orthonairovirus*	*Orthonairovirus*	Nairoviridae	ssRNA(−)	KU925488, KU925489, KU925490	Complete genome	?	1973 (Jonkers et al., 1973)	*O. maritimus* (Shi et al., 2018)	France, Great Britain, Indian Ocean, North Wales, Seychelles (Shi et al., 2018)
Tacheng tick virus 1	*Tacheng orthonairovirus*	*Orthonairovirus*	Nairoviridae	ssRNA(−)	NC_031284, NC_031285, NC_031286	Complete genome	Y (Liu et al., 2020)	2015 (Li et al., 2015)	*D. marginatus*, *D. nuttalli*, *D. silvarum*, *Hy. asiaticum* (Shi et al., 2018)	China, Kazakhstan (Shi et al., 2018)
Tamdy virus	*Tamdy orthonairovirus*	*Orthonairovirus*	Nairoviridae	ssRNA(−)	MN792651, MN792652, MN792653	Complete genome	Y (Moming et al., 2021)	2014 (L'vov et al., 2014)	*Hy. asiaticum* (L'vov et al., 1984; Moming et al., 2021)	China, Uzbekistan (Moming et al., 2021)
Tofla virus	*Tofla orthonairovirus*	*Orthonairovirus*	Nairoviridae	ssRNA(−)	NC_029124, NC_029123, NC_029122	Complete genome	?	2016 (Shimada et al., 2016)	*H. flava*, *H. formsensis* (Shimada et al., 2016)	Japan (Shimada et al., 2016)
Tunis virus	*Tunis orthonairovirus*	*Orthonairovirus*	Nairoviridae	ssRNA(−)	NC_040770, NC_040771, NC_040772	Complete genome	?	1994 (Chastel et al., 1994)	*A. hermanni* (Chastel et al., 1994)	Tunisia (Chastel et al., 1994)
Vinegar Hill virus	*Vinegar Hill orthonairovirus*	*Orthonairovirus*	Nairoviridae	ssRNA(−)	MF176881, MF176882, MF176883	Complete genome	?	2017 (Gauci et al., 2017)	*A. robertsi* (Gauci et al., 2017)	Australia (Gauci et al., 2017)
Wenzhou tick virus	*Wenzhou orthonairovirus*	*Orthonairovirus*	Nairoviridae	ssRNA(−)	NC_031291, NC_031288, NC_031289	Complete genome	?	2015 (Li et al., 2015)	*H. hystricis* (Li et al., 2015)	China (Li et al., 2015)
South Bay virus	*South Bay sabavirus*	*Sabavirus*	Nairoviridae	ssRNA(−)	KJ746878, KJ746877	Complete genome	?	2014 (Tokarz et al., 2014)	*D. nuttalli*, *H. concinna*, *H. longicornis*, *I. persulcatus*, *I. scapularis* (Tokarz et al., 2014; Zhao et al., 2021)	China, USA (Tokarz et al., 2014; Zhao et al., 2021)
Midway virus	*Midway nyavirus*	*Nyavirus*	Nyamiviridae	ssRNA(−)	NC_012702	Complete genome	?	1982 (Takahashi et al., 1982)	*Ornithodoros* spp. (Shi et al., 2018)	Central Pacific, Japan (Shi et al., 2018)
Nyamanini virus	*Nyamanini nyavirus*	*Nyavirus*	Nyamiviridae	ssRNA(−)	NC_012703	Complete genome	?	1966 (Taylor et al., 1966)	*A. arborerus*, *A. walkerae* (Shi et al., 2018)	Egypt, India, Nigeria, South Africa, Thailand (Shi et al., 2018)
Sierra Nevada virus	*Sierra Nevada nyavirus*	*Nyavirus*	Nyamiviridae	ssRNA(−)	NC_024376	Complete genome	?	2014 (Rogers et al., 2014)	*O. coriaceus* (Rogers et al., 2014)	USA (Rogers et al., 2014)
Johnston Atoll virus	*Johnston Atoll quaranjavirus*	*Quaranjavirus*	Orthomyxoviridae	ssRNA(−)	NC_052925, NC_052926, NC_052927, NC_052928, NC_052929, NC_052930, NC_052931, FJ861696, FJ861697	Complete genome	?	2009 (Presti et al., 2009)	*O. capensis* (Shi et al., 2018)	Australia, Central Pacific, New Zealand and Hawaii (Shi et al., 2018)
Quaranfil virus	*Quaranfil quaranjavirus*	*Quaranjavirus*	Orthomyxoviridae	ssRNA(−)	NC_038817, NC_038818, NC_038819, NC_038820, NC_038821, NC_038822	Complete genome	Y (Mourya et al., 2019)	1966 (Taylor et al., 1966)	*A. arboreus* (Shi et al., 2018)	Afghanistan, Egypt, Iraq, Kuwait, Nigeria, South Africa, Yemen and Iran (Shi et al., 2018)
Granville quaranjavirus^a^	*Granville quaranjavirus*	*Quaranjavirus*	Orthomyxoviridae	ssRNA(−)	MZ502307, MZ502306, MZ502305, MZ502304, MZ502308	Partial genome	?	2021 (Sameroff et al., 2021)	*Am. dissimile* (Sameroff et al., 2021)	Trinidad and Tobago (Sameroff et al., 2021)
Dhori virus	*Dhori thogotovirus*	*Thogotovirus*	Orthomyxoviridae	ssRNA(−)	NC_034254, NC_034255, NC_034256, NC_034261, NC_034262, NC_034263	Complete genome	Y (Kosoy et al., 2015)	1973 (Anderson & Casals, 1973)	*Hyalomma* spp. (Shi et al., 2018)	China, Egypt, India, Kyrgyzstan, Russia (Shi et al., 2018)
Jos virus	*Thogoto thogotovirus*	*Thogotovirus*	Orthomyxoviridae	ssRNA(−)	HM627170, HM627171, HM627172, HM627173, HM627174, HM627175	Complete genome	?	1974 (Lee et al., 1974)	*Amblyomma* spp., *Rhipicephalus* spp. (Shi et al., 2018)	Central Africa Republic, Guinea, Côte d'Ivoire, Nigeria, Senegal (Shi et al., 2018)
Thogoto virus	*Thogoto thogotovirus*	*Thogotovirus*	Orthomyxoviridae	ssRNA(−)	NC_006495, NC_006496, NC_006504, NC_006506, NC_006507, NC_006508	Complete genome	Y (Kosoy et al., 2015)	1965 (Haig et al., 1965)	*Am. variegatum*, *Boophilus* spp., *Hyalomma* spp., *Rhipicephalus* spp. (Shi et al., 2018)	Central and East Africa, Southern Europe, Southern Portugal (Shi et al., 2018)
Ngari virus	*Bunyamwera orthobunyavirus*	*Orthobunyavirus*	Peribunyaviridae	ssRNA(−)	KC608152, KC608153, KC608154	Complete genome	Y (Groseth et al., 2012)	1996 (Zeller et al., 1996)	*Am. variegatum*, *R. pulchellus* (Groseth et al., 2012)	Guinea, Kenya, Mauritania, Senegal, Somalia, Sudan (Groseth et al., 2012; Makenov et al., 2021)
Matruh virus	*Matruh orthobunyavirus*	*Orthobunyavirus*	Peribunyaviridae	ssRNA(−)	KP792693, KP792692, KP792691	Complete genome	?	1973 (Balducci et al., 1973)	*Hy. marginatum* (Balducci et al., 1973)	Italy (Balducci et al., 1973)
Bahig virus	*Tete orthobunyavirus*	*Orthobunyavirus*	Peribunyaviridae	ssRNA(−)	KP792654, KP792653, KP792652	Complete genome	?	1973 (Balducci et al., 1973)	*Hy. marginatum* (Balducci et al., 1973)	Italy (Balducci et al., 1973)
Bhanja virus	*Bhanja bandavirus*	*Bandavirus*	Phenuiviridae	ssRNA(−)	NC_027140, NC_027141, NC_027142	Complete genome	Y (Matsuno et al., 2013)	1977 (Vesenjak‐Hirjan et al., 1977)	*H. intermedia*, *R. decoloratus* (Matsuno et al., 2013)	Africa, Asia, Southern Europe, USA (Shi et al., 2018)
Heartland virus	*Heartland bandavirus*	*Bandavirus*	Phenuiviridae	ssRNA(−)	NC_024494, NC_024495, NC_024496	Complete genome	Y (McMullan et al., 2012)	2012 (McMullan et al., 2012)	*Am. americanum* (Mansfield et al., 2017)	USA (Mansfield et al., 2017)
Hunter Island virus	*Hunter Island bandavirus*	*Bandavirus*	Phenuiviridae	ssRNA(−)	NC_027715, NC_027716, NC_027717	Complete genome	?	2014 (Wang et al., 2014)	*I. eudyptidis* (Shi et al., 2018)	Australia (Shi et al., 2018)
Lone star virus	*Lone star bandavirus*	*Bandavirus*	Phenuiviridae	ssRNA(−)	NC_021242, NC_021243, NC_021244	Complete genome	?	1969 (Kokernot et al., 1969)	*Am. americanum* (Shi et al., 2018)	USA (Shi et al., 2018)
Severe fever with thrombocytopenia syndrome bunyavirus	*Dabie bandavirus*	*Bandavirus*	Phenuiviridae	ssRNA(−)	NC_043450, NC_043451, NC_043452	Complete genome	Y (Yu et al., 2011)	2011 (Yu et al., 2011)	*Dermacentor* spp., *Haemaphysalis* spp., *I. persulcatus*, *Rhipicephalus* spp. (Mansfield et al., 2017; Zhao et al., 2021)	East Asia, North America (Mansfield et al., 2017; Zhao et al., 2021)
Norway phlebovirus 1	*Norway ixovirus*	*Ixovirus*	Phenuiviridae	ssRNA(−)	NC_055433, NC_055434	Complete genome	?	2017 (Pettersson et al., 2017)	*I. ricinus* (Pettersson et al., 2017)	Norway (Pettersson et al., 2017)
Blacklegged tick phlebovirus 3	*Scapularis ixovirus*	*Ixovirus*	Phenuiviridae	ssRNA(−)	NC_055431, NC_055432	Complete genome	?	2018 (Tokarz et al., 2018)	*I. scapularis* (Tokarz et al., 2018)	USA (Tokarz et al., 2018)
Laurel Lake virus	*Laurel Lake laulavirus*	*Laulavirus*	Phenuiviridae	ssRNA(−)	NC_043679, NC_043680, NC_043681	Complete genome	?	2018 (Tokarz et al., 2018)	*I. scapularis* (Tokarz et al., 2018)	USA (Tokarz et al., 2018)
Dabieshan tick virus	*Dabieshan uukuvirus*	*Uukuvirus*	Phenuiviridae	ssRNA(−)	MW721887, MW721893	Complete genome	?	2015 (Li et al., 2015)	*H. longicornis*, *I. persulcatus*, *R. microplus* (Wang et al., 2021; Zhao et al., 2021)	China (Wang et al., 2021; Zhao et al., 2021)
Kaisodi virus	*Kaisodi uukuvirus*	*Uukuvirus*	Phenuiviridae	ssRNA(−)	NC_040492, NC_040493, NC_040494	Complete genome	?	1966 (Bhatt et al., 1966)	*H. spinigera* (Shi et al., 2018)	South India (Shi et al., 2018)
Lihan tick virus	*Lihan uukuvirus*	*Uukuvirus*	Phenuiviridae	ssRNA(−)	NC_055423, NC_055424	Complete genome	?	2015 (Li et al., 2015)	*D. nitens*, *Hy. marginatum*, *R. microplus*, *R. sanguineus* (Temmam et al., 2019)	Brazil, China, USA, Guadeloupe, Thailand, Turkey, Trinidad. Tobago (Gondard et al., 2020; Temmam et al., 2019)
Silverwater virus	*Silverwater uukuvirus*	*Uukuvirus*	Phenuiviridae	ssRNA(−)	NC_055369, NC_055370, NC_055371	Complete genome	?	1971 (Hoff et al., 1971)	*H. leporispalustris* (Shi et al., 2018)	Canada, USA (Shi et al., 2018)
Tacheng tick virus 2	*Tacheng uukuvirus*	*Uukuvirus*	Phenuiviridae	ssRNA(−)	NC_055425, NC_055426	Complete genome	Y (Dong et al., 2021)	2015 (Li et al., 2015)	*D. marginatus*, *D. reticulatus*, *R. sanguineus*, *H. marginatum* (Bratuleanu, Temmam, Chrétien, et al., 2022; Brinkmann et al., 2018; Li et al., 2015)	China, Kazakhstan, Romania, Tailand, Turkey (Bratuleanu, Temmam, Chrétien, et al., 2022; Brinkmann et al., 2018; Li et al., 2015; Temmam et al., 2019)
Uukuniemi virus	*Uukuniemi uukuvirus*	*Uukuvirus*	Phenuiviridae	ssRNA(−)	NC_005214, NC_005220, NC_005221	Complete genome	?	2013 (Palacios et al., 2013)	*I. ricinus*, *I. uriae* (Matsuno et al., 2015)	Czech Republic, Sweden, UK (Matsuno et al., 2015)
Yongjia tick virus 1	*Yongjia uukuvirus*	*Uukuvirus*	Phenuiviridae	ssRNA(−)	NC_055427, NC_055428	Complete genome	?	2015 (Li et al., 2015)	*H. hystricis* (Li et al., 2015)	China (Li et al., 2015)
Blanchseco virus	*Blanchseco alpharicinrhavirus*	*Alpharicinrhavirus*	Rhabdoviridae	ssRNA(−)	MN025503	Complete genome	?	2019 (Sameroff et al., 2019)	*A. ovale* (Sameroff et al., 2019)	Trinidad and Tobago (Sameroff et al., 2019)
Bole tick virus 2	*Bole alpharicinrhavirus*	*Alpharicinrhavirus*	Rhabdoviridae	ssRNA(−)	NC_031079	Complete genome	?	2015 (Li et al., 2015)	*Hy. asiaticum* (Li et al., 2015)	China (Li et al., 2015)
Wuhan tick virus 1	*Wuhan alpharicinrhavirus*	*Alpharicinrhavirus*	Rhabdoviridae	ssRNA(−)	NC_031304	Complete genome	?	2015 (Li et al., 2015)	*D. marginatus*, *R. microplus*, *R. annulatus*, *H. longicornis* (Temmam et al., 2019; Xu et al., 2021)	China, Turkey (Xu et al., 2021)
New Kent County virus	*Kent ephemerovirus*	*Ephemerovirus*	Rhabdoviridae	ssRNA(−)	MF615270	Complete genome	?	2018 (Tokarz et al., 2018)	*I. scapularis (*Tokarz et al., 2018 *)*	USA (Tokarz et al., 2018)
Barur virus	*Barur ledantevirus*	*Ledantevirus*	Rhabdoviridae	ssRNA(−)	NC_034535	Complete genome	?	1981 (Butenko et al., 1981)	*Hy. intermedia* (Shi et al., 2018)	India, Kenya, Somalia (Shi et al., 2018)
Kolente virus	*Kolente ledantevirus*	*Ledantevirus*	Rhabdoviridae	ssRNA(−)	NC_025342	Complete genome	?	2013 (Ghedin et al., 2013)	*Am. variegatum* (Shi et al., 2018)	Guinea (Shi et al., 2018)
Yongjia tick virus 2	*Yongjia ledantevirus*	*Ledantevirus*	Rhabdoviridae	ssRNA(−)	NC_031305	Complete genome	?	2015 (Li et al., 2015)	*H. hystricis* (Shi et al., 2018)	China (Shi et al., 2018)
Connecticut virus	*Connecticut sawgrhavirus*	*Sawgrhavirus*	Rhabdoviridae	ssRNA(−)	KM205020	Complete genome	?	1980 (Main & Carey, 1980)	*I. dentatus* (Shi et al., 2018)	USA (Shi et al., 2018)
Long Island tick rhabdovirus	*Island sawgrhavirus*	*Sawgrhavirus*	Rhabdoviridae	ssRNA(−)	NC_025340	Complete genome	?	2014 (Tokarz et al., 2014)	*Am. americanum* (Tokarz et al., 2014)	USA (Tokarz et al., 2014)
New Minto virus	*Minto sawgrhavirus*	*Sawgrhavirus*	Rhabdoviridae	ssRNA(−)	NC_055457	Complete genome	?	1978 (Ritter et al., 1978)	*H. ieporispalustris* (Shi et al., 2018)	USA (Shi et al., 2018)
Sawgrass virus	*Sawgrass sawgrhavirus*	*Sawgrhavirus*	Rhabdoviridae	ssRNA(−)	NC_055461	Complete genome	?	1970 (Sather et al., 1970)	*D. variabilis*, *H. ieporispalustris* (Shi et al., 2018)	USA (Shi et al., 2018)
Isfahan virus	*Isfahan vesiculovirus*	*Vesiculovirus*	Rhabdoviridae	ssRNA(−)	NC_020806	Complete genome	?	1977 (Tesh et al., 1977)	*Hy. asiaticum* (Shi et al., 2018)	Parts of Asia, Turkmenistan (Shi et al., 2018)
Zahedan rhabdovirus	*Zahedan zarhavirus*	*Zarhavirus*	Rhabdoviridae	ssRNA(−)	NC_040664	Complete genome	?	2015 (Dilcher et al., 2015)	*Hy. anatolicum* (Shi et al., 2018)	Iran (Shi et al., 2018)
Beiji nairovirus	Unclassified	*Nairovirus*	Nairoviridae	ssRNA(−)	MW315106, MW315112	Partial genome	Y (Wang et al., 2021)	2019 (Meng et al., 2019)	*I. crenulatus*, *I. persulcatus* (Wang et al., 2021)	China (Wang et al., 2021)
Nayun tick nairovirus	Unclassified	*Nairovirus*	Nairoviridae	ssRNA(−)	MW561151, KP141756, KP141755	Partial genome	?	2015 (Xia et al., 2015)	*R. sanguineus* (Bratuleanu, Temmam, Chrétien, et al., 2022; Xia et al., 2015)	China, Romania (Bratuleanu, Temmam, Chrétien, et al., 2022; Xia et al., 2015)
Norway nairovirus 1	Unclassified	*Nairovirus*	Nairoviridae	ssRNA(−)	MF141045, MF141044	Partial genome	?	2017 (Pettersson et al., 2017)	*I. ricinus* (Pettersson et al., 2017)	Northern Europe (Pettersson et al., 2017)
Paramushir virus	Unclassified	*Nairovirus*	Nairoviridae	ssRNA(−)	MH638289, MH124638, MH124637, MH124636, MH124635, MH124634, KF801657, KP792719, KP792718, KP792717	Partial genome	?	1976 (L'vov et al., 1976)	*I. signatus*, *I. uriae* (L'vov et al., 1976)	Russia (Safonova et al., 2019)
Rondonia orthonairovirus	Unclassified	*Orthonairovirus*	Nairoviridae	ssRNA(−)	MN560621, MN560622, MN560623, MN560624, MN560625, MN560626, MN560627, MN560628, MN560629	Partial genome	?	2019 (Blomström et al., 2019)	*Antricola* spp. (Blomström et al., 2019)	Brazil (Blomström et al., 2019)
Songling virus	Unclassified	*Orthonairovirus*	Nairoviridae	ssRNA(−)	MT328780, MT328779, MT328778	Partial genome	Y (Ma et al., 2021)	2021 (Ma et al., 2021)	*H. longicornis* (Ma et al., 2021)	China (Ma et al., 2021)
Yezo virus	Unclassified	*Orthonairovirus*	Nairoviridae	ssRNA(−)	LC621360, LC621359, LC621358	Partial genome	Y (Kodama et al., 2021)	2021 (Kodama et al., 2021)	*H. megaspinosa* (Kodama et al., 2021)	Japan (Kodama et al., 2021)
Zambezi tick virus 1	Unclassified	*Quarjavirus*	Orthomyxoviridae	ssRNA(−)	MH267793	Partial genome	?	2018 (Cholleti et al., 2018)	*Rhipicephalus* spp. (Cholleti et al., 2018)	Mozambique (Cholleti et al., 2018)
Aransas Bay virus	Unclassified	*Thogotovirus*	Orthomyxoviridae	ssRNA(−)	KC506162, KC506163, KC506164, KC506165, KC506166, KC506167	Partial genome	?	1979 (Yunker et al., 1979)	*Ornithodoros* spp. (Briese et al., 2014)	North America (Briese et al., 2014)
Bourbon virus	Unclassified	*Thogotovirus*	Orthomyxoviridae	ssRNA(−)	KU708253, KU708254, KU708255, MH880287, MH880288, MH880289, MH880290, MH880291, MH880292	Complete genome	Y (Kosoy et al., 2015)	2015 (Kosoy et al., 2015)	*Am. americanum* (Savage et al., 2017)	USA (Kosoy et al., 2015)
Oz virus	Unclassified	*Thogotovirus*	Orthomyxoviridae	ssRNA(−)	NC_040730, NC_040731, NC_040732, NC_040733, NC_040734, NC_040735	Complete genome	?	2018 (Ejiri et al., 2018)	*Am. testudinarium* (Ejiri et al., 2018)	Japan (Ejiri et al., 2018)
Thailand tick thogotovirus	Unclassified	*Thogotovirus*	Orthomyxoviridae	ssRNA(−)	MN095539, MN095540, MN095541, MN095542, MN095543, MN095544	Complete genome	?	2019 (Temmam et al., 2019)	*R. microplus* (Temmam et al., 2019)	Tailand (Temmam et al., 2019)
Upolu virus	Unclassified	*Thogotovirus*	Orthomyxoviridae	ssRNA(−)	KC506156, KC506157, KC506158, KC506159, KC506160, KC506161	Complete genome	?	1968 (Doherty et al., 1969)	*O. capensis* (Briese et al., 2014)	Australia (Briese et al., 2014)
American dog tick phlebovirus	Unclassified	*Phlebovirus*	Phenuiviridae	ssRNA(−)	KM048312, KM048311	Complete genome	?	2014 (Tokarz et al., 2014)	*D. variabilis* (Tokarz et al., 2014)	USA (Tokarz et al., 2014)
Blacklegged tick phlebovirus 1	Unclassified	*Phlebovirus*	Phenuiviridae	ssRNA(−)	KX184200, KX184201	Complete genome	?	2014 (Tokarz et al., 2014)	*I. scapularis* (Tokarz et al., 2014)	USA (Tokarz et al., 2014)
Blacklegged tick phlebovirus 2	Unclassified	*Phlebovirus*	Phenuiviridae	ssRNA(−)	KM589357, KM589354	Partial genome	?	2014 (Tokarz et al., 2014)	*I. scapularis* (Tokarz et al., 2014)	USA (Tokarz et al., 2014)
Bole tick virus 1	Unclassified	*Phlebovirus*	Phenuiviridae	ssRNA(−)	MZ244253, MZ244252, KM817731, MH916622, MH916621, MH916620, MH916619, MH688504, MH836620, MH688505, MH688503, MH688501, MH688578, MH688577, MH688576, MH688575, MH688574, MH688573, MH688572, MH688571	Partial genome	?	2015 (Li et al., 2015)	*Hy. asiaticum* (Li et al., 2015)	China (Li et al., 2015)
Brown dog tick phlebovirus 1	Unclassified	*Phlebovirus*	Phenuiviridae	ssRNA(−)	MZ244261, MZ244260, MZ244259, MZ244256, MN025507, MN025506	Partial genome	?	2019 (Sameroff et al., 2019)	*R. sanguineus*, *R. microplus* (Sameroff et al., 2019)	China, Trinidad and Tobago (Sameroff et al., 2019)
Brown dog tick phlebovirus 2	Unclassified	*Phlebovirus*	Phenuiviridae	ssRNA(−)	MW561139, MW561138, MW561137, MW561136, MN025509, MN025508	Partial genome	?	2019 (Sameroff et al., 2019)	*R. microplus*, *R. sanguineus* (Sameroff et al., 2019)	China, Trinidad and Tobago (Sameroff et al., 2019)
Changping tick virus 1	Unclassified	*Phlebovirus*	Phenuiviridae	ssRNA(−)	MW561146, MW561145, MW561144, MW561143, MW561142, MW561141, MW561140, KM817732, KM817665	Partial genome	?	2015 (Li et al., 2015)	*Dermacentor* spp. (Li et al., 2015)	China, Romania (Li et al., 2015; Matsuno et al., 2013)
Lanjan virus	Unclassified	*Phlebovirus*	Phenuiviridae	ssRNA(−)	KM370974	Partial genome	?	1967 (Tan et al., 1967)	*D. auratus* (Shi et al., 2018)	Malaysia (Shi et al., 2018)
Palma virus	Unclassified	*Phlebovirus*	Phenuiviridae	ssRNA(−)	JX961630, JX961629, JX961628	Partial genome	?	1994 (Filipe et al., 1994)	*H. punctate* (Shi et al., 2018)	Portugal (Shi et al., 2018)
Rhipicephalus associated phlebovirus 1	Unclassified	*Phlebovirus*	Phenuiviridae	ssRNA(−)	MZ244255, MZ244254, MH814976, MH814975	Partial genome	?	2021 (Shi et al., 2021)	*R. microplus* (Shi et al., 2021)	China (Shi et al., 2021)
Tick phlebovirus Anatolia 1	Unclassified	*Phlebovirus*	Phenuiviridae	ssRNA(−)	MG764522, MG764521	Partial genome	?	2018 (Brinkmann et al., 2018)	*R. sanguineus* (Brinkmann et al., 2018)	Turkey (Brinkmann et al., 2018)
Neke harbour virus	Unclassified	Unclassified	Orthomyxoviridae	ssRNA(−)	MN830238	Partial genome	?	2020 (Pettersson et al., 2020)	*I. uriae* (Pettersson et al., 2020)	Antarctica (Pettersson et al., 2020)
Uumaja virus	Unclassified	Unclassified	Orthomyxoviridae	ssRNA(−)	MN830237	Partial genome	?	2020 (Pettersson et al., 2020)	*I. uriae* (Pettersson et al., 2020)	Northern Sweden (Pettersson et al., 2020)
Khasan virus	Unclassified	Unclassified	Peribunyaviridae	ssRNA(−)	KF892046, KF892047, KF892048	Partial genome	?	1978 (L'vov et al., 1978)	*H. longgicornis* (Shi et al., 2018)	Russia (Shi et al., 2018)
American dog tick rhabdovirus 2	Unclassified	Unclassified	Rhabdoviridae	ssRNA(−)	MF962659, MF962661	Partial genome	?	2018 (Tokarz et al., 2018)	*I. scapularis* (Tokarz et al., 2018)	USA (Tokarz et al., 2018)
Blacklegged tick rhabdovirus 1	Unclassified	Unclassified	Rhabdoviridae	ssRNA(−)	MF360790	Complete genome	?	2018 (Tokarz et al., 2018)	*I. scapularis* (Tokarz et al., 2018)	USA (Tokarz et al., 2018)
Dog tick rhabdovirus 1	Unclassified	Unclassified	Rhabdoviridae	ssRNA(−)	MF360791	Partial genome	?	2018 (Tokarz et al., 2018)	*I. scapularis (*Tokarz et al., 2018 *)*	USA (Tokarz et al., 2018)
Huangpi tick virus 3	Unclassified	Unclassified	Rhabdoviridae	ssRNA(−)	NC_031083	Complete genome	?	2015 (Li et al., 2015)	*H. doenitzi* (Li et al., 2015)	China (Li et al., 2015)
Nayun tick rhabdovirus	Unclassified	Unclassified	Rhabdoviridae	ssRNA(−)	KP141757	Partial genome	?	2015 (Xia et al., 2015)	*Rhipicephalus* spp. (Xia et al., 2015)	China (Xia et al., 2015)
Rhipicephalus associated rhabdo‐like virus	Unclassified	Unclassified	Rhabdoviridae	ssRNA(−)	MH814974	Partial genome	?	2021 (Shi et al., 2021)	*H. longicornis*, *R. microplus* (Shi et al., 2021)	China (Shi et al., 2021)
Tacheng tick virus 3	Unclassified	Unclassified	Rhabdoviridae	ssRNA(−)	NC_031268	Complete genome	?	2015 (Li et al., 2015)	*D. marginatus* (Li et al., 2015)	China (Li et al., 2015)
Tacheng tick virus 7	Unclassified	Unclassified	Rhabdoviridae	ssRNA(−)	NC_031272	Complete genome	?	2015 (Li et al., 2015)	*A. miniatus* (Li et al., 2015)	China (Li et al., 2015)
Taishun tick virus	Unclassified	Unclassified	Rhabdoviridae	ssRNA(−)	NC_031273	Complete genome	?	2015 (Li et al., 2015)	*H. hystricis* (Li et al., 2015)	China, Russia (Li et al., 2015)
Deer tick mononegavirales‐like virus	Unclassified	Unclassified	Unclassified	ssRNA(−)	MG880117, KM048317, KJ746903	Partial genome	?	2014 (Tokarz et al., 2014)	*D. nuttalli*, *D. silvarum*, *H. concinna*, *I. persulcatus*, *I. scapularis* (Tokarz et al., 2014; Zhao et al., 2021)	China, USA (Tokarz et al., 2014; Zhao et al., 2021)
Bonden virus	Unclassified	Unclassified	Unclassified Bunyavirales	ssRNA(−)	MN830227	Partial genome	?	2020 (Pettersson et al., 2020)	*I. uriae* (Pettersson et al., 2020)	Northern Sweden (Pettersson et al., 2020)
Bronnoya virus	Unclassified	Unclassified	Unclassified Bunyavirales	ssRNA(−)	MF141063, MF141062	Partial genome	?	2017 (Pettersson et al., 2017)	*I. ricinus* (Pettersson et al., 2017)	Norway (Pettersson et al., 2017)
Ubmeje virus	Unclassified	Unclassified	Unclassified Bunyavirales	ssRNA(−)	MN830232	Partial genome	?	2020 (Pettersson et al., 2020)	*I. uriae* (Pettersson et al., 2020)	Northern Sweden (Pettersson et al., 2020)
Norway mononegavirus 1	Unclassified	Unclassified	Unclassified Mononegavirales	ssRNA(−)	MF141072	Partial genome	?	2017 (Pettersson et al., 2017)	*I. ricinus* (Pettersson et al., 2017)	Norway (Pettersson et al., 2017)
Tick mononegavirus	Unclassified	Unclassified	Unclassified Mononegavirales	ssRNA(−)	MZ244310, MH835441, MH835440, MH835439, MH835438	Partial genome	?	2018 (Yang et al., 2018)	*I. scapularis*, *H. longicornis* (Yang et al., 2018)	China (Yang et al., 2018)
Umea virus	Unclassified	Unclassified	Unclassified Mononegavirales	ssRNA(−)	MN830236	Partial genome	?	2020 (Pettersson et al., 2020)	*I. uriae* (Pettersson et al., 2020)	Sweden (Pettersson et al., 2020)
Cannepointvirus	Unclassified	Unclassified	Unclassified Riboviria	ssRNA(−)	MK026566	Partial genome	?	2018 (Harvey et al., 2019)	*Am. moreliae* (Harvey et al., 2019)	Australia (Harvey et al., 2019)
Fairlight virus	Unclassified	Unclassified	Unclassified Riboviria	ssRNA(−)	MK026565	Partial genome	?	2018 (Harvey et al., 2019)	*Am. moreliae* (Harvey et al., 2019)	Australia (Harvey et al., 2019)
Manly virus	Unclassified	Unclassified	Unclassified Riboviria	ssRNA(−)	MK026564	Partial genome	?	2018 (Harvey et al., 2019)	*Am. moreliae* (Harvey et al., 2019)	Australia (Harvey et al., 2019)
Messner virus	Unclassified	Unclassified	Unclassified Riboviria	ssRNA(−)	MT025174	Partial genome	?	2020 (Wille et al., 2020)	*I. uriae* (Wille et al., 2020)	Antarctica (Wille et al., 2020)
North Shore virus	Unclassified	Unclassified	Unclassified Riboviria	ssRNA(−)	OL452126	Partial genome	?	2018 (Harvey et al., 2019)	*I. holocyclus* (Harvey et al., 2019)	Australia (Harvey et al., 2019)
Old Quarry Swamp virus	Unclassified	Unclassified	Unclassified Riboviria	ssRNA(−)	MK026595, MK026596, MK026597, MK026598, MK026599	Partial genome	?	2018 (Harvey et al., 2019)	*I. holocyclus* (Harvey et al., 2019)	Australia (Harvey et al., 2019)
Piguzov virus	Unclassified	Unclassified	Unclassified Riboviria	ssRNA(−)	MT025176, MT025177	Partial genome	?	2020 (Pettersson et al., 2020)	*I. uriae* (Pettersson et al., 2020)	Antarctica (Pettersson et al., 2020)
Quarantine Head virus	Unclassified	Unclassified	Unclassified Riboviria	ssRNA(−)	MK026568	Partial genome	?	2018 (Harvey et al., 2019)	*Am. moreliae* (Harvey et al., 2019)	Australia (Harvey et al., 2019)
Ronne virus	Unclassified	Unclassified	Unclassified Riboviria	ssRNA(−)	MT025165, MT025164	Partial genome	?	2020 (Pettersson et al., 2020; Wille et al., 2020)	*I. uriae* (Pettersson et al., 2020; Wille et al., 2020)	Antarctica, Northern Sweden (Pettersson et al., 2020; Wille et al., 2020)
Tacheng tick virus 5	Unclassified	Unclassified	Unclassified Riboviria	ssRNA(−)	NC_028264	Complete genome	?	2015 (Li et al., 2015)	*D. marginatus* (Li et al., 2015)	China, Kazakhstan (Li et al., 2015)
Timbillica virus	Unclassified	Unclassified	Unclassified Riboviria	ssRNA(−)	MK026589, MK026590	Partial genome	?	2018 (Harvey et al., 2019)	*I. holocyclus* (Harvey et al., 2019)	Australia (Harvey et al., 2019)
Gadgets Gully virus	*Gadgets Gully virus*	*Flavivirus*	Flaviviridae	ssRNA(+)	NC_033723	Complete genome	?	1985 (St George et al., 1985)	*I. uriae* (Pettersson et al., 2020)	Antarctica, Australia (Pettersson et al., 2020)
Kadam virus	*Kadam virus*	*Flavivirus*	Flaviviridae	ssRNA(+)	NC_033724	Complete genome	?	1970 (Henderson et al., 1970)	*R. pravus* (Shi et al., 2018)	Saudi Arabia, Uganda (Shi et al., 2018)
Alkhumra hemorrhagic fever virus	*Kyasanur Forest disease virus*	*Flavivirus*	Flaviviridae	ssRNA(+)	AF331718	Complete genome	Y (Grard et al., 2007)	1957 (Work et al., 1957)	*H. spinigera*, *O. savignyi* (Shi et al., 2018)	India, Saudi Arabia (Shi et al., 2018)
Langat virus	*Langat virus*	*Flavivirus*	Flaviviridae	ssRNA(+)	AF253419	Complete genome	?	1956 (Gordon Smith, 1956)	*I. granulatus* (Shi et al., 2018)	Malaysia (Shi et al., 2018)
Louping ill virus	*Louping ill virus*	*Flavivirus*	Flaviviridae	ssRNA(+)	Y07863	Complete genome	Y (Davidson et al., 1991)	1931 (Greig et al., 1931)	*I. ricinus* (Shi et al., 2018)	England, Ireland, Scotland, Wales (Shi et al., 2018)
Meaban virus	*Meaban virus*	*Flavivirus*	Flaviviridae	ssRNA(+)	NC_033721	Complete genome	?	1985 (Chastel et al., 1985)	*O. maritimus* (Shi et al., 2018)	France (Shi et al., 2018)
Omsk hemorrhagic fever virus	*Omsk hemorrhagic fever virus*	*Flavivirus*	Flaviviridae	ssRNA(+)	AY193805	Complete genome	Y (Grard et al., 2007)	1989 (Gaĭdamovich et al., 1989)	*Dermacentor* spp., *I. persculatus* (Růžek et al., 2010)	Russia (Grard et al., 2007)
Deer tick virus	*Powassan virus*	*Flavivirus*	Flaviviridae	ssRNA(+)	AF311056	Complete genome	?	1997 (Telford 3rd et al., 1997)	*I. scapularis* (Mansfield et al., 2017; Shi et al., 2018)	New England, North America (Mansfield et al., 2017; Shi et al., 2018)
Powassan virus	*Powassan virus*	*Flavivirus*	Flaviviridae	ssRNA(+)	L06436	Complete genome	Y (Tokarz et al., 2014)	1959 (McLean & Donohue, 1959)	*I. cookei*, *Ixodes* spp. (Tokarz et al., 2014)	North America, Russia (Mansfield et al., 2017)
Karshi virus	*Royal Farm virus*	*Flavivirus*	Flaviviridae	ssRNA(+)	AY863002	Complete genome	?	1976 (L'vov et al., 1976)	*A. hermanni*, *O. papillipes* (Shi et al., 2018)	North of Central Asia, Uzbek (Shi et al., 2018)
Saumarez Reef virus	*Saumarez Reef virus*	*Flavivirus*	Flaviviridae	ssRNA(+)	DQ235150	Complete genome	?	1977 (St George et al., 1977)	*I. eudyptidis*, *O. capensis* (Shi et al., 2018)	Australia (Shi et al., 2018)
Tick‐borne encephalitis virus	*Tick‐borne encephalitis virus*	*Flavivirus*	Flaviviridae	ssRNA(+)	NC_001672	Complete genome	Y (Shi et al., 2018)	1931 (Schneider, 1931)	*I. ovatus*, *I. persulcatus*, *I. ricinus* (Shi et al., 2018)	Northern Asia, Northern Europe, Siberia (Shi et al., 2018)
Tyuleniy virus	*Tyuleniy virus*	*Flavivirus*	Flaviviridae	ssRNA(+)	NC_023424	Complete genome	?	1971 (L'vov et al., 1971)	*I. putus* (Shi et al., 2018)	Tuleniy Island (Shi et al., 2018)
Ixodes holocyclus iflavirus	*Ixodes holocyclus iflavirus*	*Iflavirus*	Iflaviridae	ssRNA(+)	OL452115	Partial genome	?	2018 (O'Brien et al., 2018)	*I. holocyclus* (O'Brien et al., 2018)	Australia (O'Brien et al., 2018)
Blacklegged tick associated ilarvirus	Unclassified	*Ilarvirus*	Bromoviridae	ssRNA(+)	MG647776, MG647777	Partial genome	?	2018 (Tokarz et al., 2018)	*I. scapularis* (Tokarz et al., 2018)	USA (Tokarz et al., 2018)
Trinbago virus	Unclassified	*Pestivirus*	Flaviviridae	ssRNA(+)	MN025505	Partial genome	?	2019 (Sameroff et al., 2019)	*A. ovale*, *R. microplus*, *R. sanguineus* (Sameroff et al., 2019)	Trinidad, Tobago (Sameroff et al., 2019)
Cattle tick tymovirus‐like virus 1	Unclassified	*Tymovirus*	Tymoviridae	ssRNA(+)	MN025504	Partial genome	?	2019 (Sameroff et al., 2019)	*R. microplus* (Sameroff et al., 2019)	Trinidad and Tobago (Sameroff et al., 2019)
Lone star tick dicistrovirus	Unclassified	Unclassified	Dicistroviridae	ssRNA(+)	KX774633	Partial genome	?	2018 (Tokarz et al., 2018)	*I. scapularis* (Tokarz et al., 2018)	USA (Tokarz et al., 2018)
Alongshan virus	Unclassified	Unclassified	Flaviviridae	ssRNA(+)	MN648770, MN648771, MN648772, MN648773	Partial genome	Y (Wang et al., 2019)	2019 (Kuivanen et al., 2019)	*I. persulcatus*, *I. ricinus* (Kholodilov et al., 2020)	China, France, Finland (Kholodilov et al., 2020)
Jingmen tick virus	Unclassified	Unclassified	Flaviviridae	ssRNA(+)	MK721629, MK721630, MK721631, MK721632	Complete genome	Y (Jia et al., 2019)	2014 (Qin et al., 2014)	*Am. javanense*, *D. nuttalli*, *Haemaphysalis* spp., *Ixodes* spp., *Rhipicephalus* spp. (Zhao et al., 2021)	Many countries in Asia, America, and Africa (Vandegrift et al., 2020; Zhao et al., 2021)
Norway luteo‐like virus 1	Unclassified	Unclassified	Luteoviridae	ssRNA(+)	MF141065	Partial genome	?	2017 (Pettersson et al., 2017)	*I. ricinus* (Pettersson et al., 2017)	Norway (Pettersson et al., 2017)
Norway luteo‐like virus 2	Unclassified	Unclassified	Luteoviridae	ssRNA(+)	MF141068	Partial genome	?	2017 (Pettersson et al., 2017)	*I. ricinus* (Pettersson et al., 2017)	Norway (Pettersson et al., 2017)
Norway luteo‐like virus 3	Unclassified	Unclassified	Luteoviridae	ssRNA(+)	MF141069	Partial genome	?	2017 (Pettersson et al., 2017)	*I. ricinus* (Pettersson et al., 2017)	Norway (Pettersson et al., 2017)
Norway luteo‐like virus 4	Unclassified	Unclassified	Luteoviridae	ssRNA(+)	MF141070	Partial genome	?	2017 (Pettersson et al., 2017)	*I. ricinus* (Pettersson et al., 2017)	Norway (Pettersson et al., 2017)
Lone star tick nodavirus	Unclassified	Unclassified	Nodaviridae	ssRNA(+)	KX774635, KX774634	Partial genome	?	2018 (Tokarz et al., 2018)	*I. scapularis* (Tokarz et al., 2018)	USA (Tokarz et al., 2018)
Paradise bay virus	Unclassified	Unclassified	Tombusviridae	ssRNA(+)	MN830247	Partial genome	?	2020 (Pettersson et al., 2020)	*I. uriae* (Pettersson et al., 2020)	Antarctica (Pettersson et al., 2020)
Upmeje virus	Unclassified	Unclassified	Tombusviridae	ssRNA(+)	MN830246	Partial genome	?	2020 (Pettersson et al., 2020)	*I. uriae*(Pettersson et al., 2020)	Northern Sweden (Pettersson et al., 2020)
Bagotville virus	Unclassified	Unclassified	Unclassified Picornavirales	ssRNA(+)	MW741892	Partial genome	?	2021 (Chandra et al., 2021)	*I. holocyclus* (Chandra et al., 2021)	Australia (Chandra et al., 2021)
Blacklegged tick picorna‐like virus 1	Unclassified	Unclassified	Unclassified Picornavirales	ssRNA(+)	MG647769, MG647774	Partial genome	?	2018 (Tokarz et al., 2018)	*I. scapularis* (Tokarz et al., 2018)	USA (Tokarz et al., 2018)
Blacklegged tick picorna‐like virus 2	Unclassified	Unclassified	Unclassified Picornavirales	ssRNA(+)	MG647773, MG647772, MG647771, MG647770	Partial genome	?	2018 (Tokarz et al., 2018)	*I. scapularis* (Tokarz et al., 2018)	USA (Tokarz et al., 2018)
Wardell virus	Unclassified	Unclassified	Unclassified Picornavirales	ssRNA(+)	MW741896, MW741895, MW741894	Partial genome	?	2021 (Chandra et al., 2021)	*I. holocyclus* (Chandra et al., 2021)	Australia (Chandra et al., 2021)
Woodburn virus	Unclassified	Unclassified	Unclassified Picornavirales	ssRNA(+)	MW741893	Partial genome	?	2021 (Chandra et al., 2021)	*I. holocyclus* (Chandra et al., 2021)	Australia (Chandra et al., 2021)
Blue Fish Point virus	Unclassified	Unclassified	Unclassified Riboviria	ssRNA(+)	MK026570, MK026571	Partial genome	?	2018 (Harvey et al., 2019)	*I. holocyclus* (Harvey et al., 2019)	Australia (Harvey et al., 2019)
Bole tick virus 4	Unclassified	Unclassified	Unclassified Riboviria	ssRNA(+)	MZ244286	Complete genome	?	2015 (Shi et al., 2015)	*D. marginatus*, *D. reticulatus*, *H. asiaticum*, *Hy. asiaticum*, *Hy. detritum*, *R. microplus*, *R. sanguineus* (Bratuleanu, Temmam, Chrétien, et al., 2022; Temmam et al., 2019; Zakham et al., 2021)	China, Romania, Saudi Arabia, Thailand, Trinidad, Tobago (Bratuleanu, Temmam, Chrétien, et al., 2022; Temmam et al., 2019; Zakham et al., 2021)
Bulatov virus	Unclassified	Unclassified	Unclassified Riboviria	ssRNA(+)	MN830234, MT025173	Partial genome	?	2020 (Pettersson et al., 2020; Wille et al., 2020)	*I. uriae* (Pettersson et al., 2020)	Antarctica (Pettersson et al., 2020)
Collins Beach virus	Unclassified	Unclassified	Unclassified Riboviria	ssRNA(+)	MK026585	Partial genome	?	2018 (Harvey et al., 2019)	*I. holocyclus* (Harvey et al., 2019)	Australia (Harvey et al., 2019)
Fairfax Lookout virus	Unclassified	Unclassified	Unclassified Riboviria	ssRNA(+)	MK026588, MK026587, MK026586	Partial genome	?	2018 (Harvey et al., 2019)	*I. holocyclus* (Harvey et al., 2019)	Australia (Harvey et al., 2019)
Gerbovich virus	Unclassified	Unclassified	Unclassified Riboviria	ssRNA(+)	MN830239, MT025175	Partial genome	?	2020 (Pettersson et al., 2020; Wille et al., 2020)	*I. uriae* (Pettersson et al., 2020; Wille et al., 2020)	Antarctica (Pettersson et al., 2020; Wille et al., 2020)
Ingleside virus	Unclassified	Unclassified	Unclassified Riboviria	ssRNA(+)	OL452105	Partial genome	?	2018 (Harvey et al., 2019)	*I. holocyclus* (Harvey et al., 2019)	Australia (Harvey et al., 2019)
Ixodes scapularis associated virus 3	Unclassified	Unclassified	Unclassified Riboviria	ssRNA(+)	LC094965, MG677814	Partial genome	?	2017 (Nakao et al., 2017)	*I. scapularis* (Nakao et al., 2017; Tokarz et al., 2018)	USA, Japan (Nakao et al., 2017; Tokarz et al., 2018)
Jump Rock virus	Unclassified	Unclassified	Unclassified Riboviria	ssRNA(+)	MK026583	Partial genome	?	2018 (Harvey et al., 2019)	*I. holocyclus* (Harvey et al., 2019)	Australia (Harvey et al., 2019)
Nadgee virus	Unclassified	Unclassified	Unclassified Riboviria	ssRNA(+)	MK026592	Partial genome	?	2018 (Harvey et al., 2019)	*I. holocyclus* (Harvey et al., 2019)	Australia (Harvey et al., 2019)
Store Beach virus	Unclassified	Unclassified	Unclassified Riboviria	ssRNA(+)	MK026567	Partial genome	?	2018 (Harvey et al., 2019)	*Am. moreliae* (Harvey et al., 2019)	Australia (Harvey et al., 2019)
Tacheng tick virus 8	Unclassified	Unclassified	Unclassified Riboviria	ssRNA(+)	NC_028367	Complete genome	?	2015 (Shi et al., 2015)	*D. marginatus* (Shi et al., 2015)	China (Shi et al., 2015)
Vovk virus	Unclassified	Unclassified	Unclassified Riboviria	ssRNA(+)	MN830235, MT025162	Partial genome	?	2020 (Pettersson et al., 2020; Wille et al., 2020)	*I. uriae* (Pettersson et al., 2020; Wille et al., 2020)	Antarctica (Pettersson et al., 2020; Wille et al., 2020)
Wangarabell virus	Unclassified	Unclassified	Unclassified Riboviria	ssRNA(+)	MK026593	Partial genome	?	2018 (Harvey et al., 2019)	*I. holocyclus* (Harvey et al., 2019)	Australia (Harvey et al., 2019)
Yambulla virus	Unclassified	Unclassified	Unclassified Riboviria	ssRNA(+)	MK026594	Partial genome	?	2018 (Harvey et al., 2019)	*I. holocyclus* (Harvey et al., 2019)	Australia (Harvey et al., 2019)
Antioquia tymovirus‐like 1	Unclassified	Unclassified	Unclassified Tymovirales	ssRNA(+)	MK683451	Partial genome	?	2020 (Gómez et al., 2020)	*R. microplus* (Gómez et al., 2020)	Colombia (Gómez et al., 2020)
Guarapuava tymovirus‐like 1	Unclassified	Unclassified	Unclassified Tymovirales	ssRNA(+)	MH155882	Partial genome	?	2018 (de Souza et al., 2018)	*R. microplus* (de Souza et al., 2018)	Brazil (de Souza et al., 2018)
American dog tick associated virus 1	Unclassified	Unclassified	Unclassified	Unknown	MF962660	Partial genome	?	2018 (Tokarz et al., 2018)	*D. variabilis* (Tokarz et al., 2018)	USA (Tokarz et al., 2018)
American dog tick associated virus 2	Unclassified	Unclassified	Unclassified	Unknown	MF962662, MF962657	Partial genome	?	2018 (Tokarz et al., 2018)	*D. variabilis* (Tokarz et al., 2018)	USA (Tokarz et al., 2018)
Finch Creek virus	Unclassified	Unclassified	Unclassified	Unknown	EU267169	Partial genome	?	2009 (Major et al., 2009)	*I. uriae* (Major et al., 2009)	Australia (Major et al., 2009)
Ganjam virus	Unclassified	Unclassified	Unclassified	Unknown	KU925466, KU925465, KU925464	Partial genome	?	1969 (Dandawate & Shah, 1969)	*H. intermedia* (Kuhn et al., 2016)	India (Kuhn et al., 2016)
Ixodes scapularis associated virus 1	Unclassified	Unclassified	Unclassified	Unknown	LC094964, KM048318	Partial genome	?	2014 (Tokarz et al., 2014)	*I. scapularis* (Nakao et al., 2017; Tokarz et al., 2014)	Japan, USA (Nakao et al., 2017; Tokarz et al., 2014)
Ixodes scapularis associated virus 2	Unclassified	Unclassified	Unclassified	Unknown	KM048319	Partial genome	?	2014 (Tokarz et al., 2014)	*I. scapularis* (Tokarz et al., 2014)	USA (Tokarz et al., 2014)
Ixodes scapularis associated virus 4	Unclassified	Unclassified	Unclassified	Unknown	MF962656	Partial genome	?	2018 (Tokarz et al., 2018)	*I. scapularis* (Tokarz et al., 2018)	USA (Tokarz et al., 2018)
Ixodes scapularis associated virus 5	Unclassified	Unclassified	Unclassified	Unknown	MG256513	Partial genome	?	2018 (Tokarz et al., 2018)	*I. scapularis* (Tokarz et al., 2018)	USA (Tokarz et al., 2018)
Ixodes scapularis associated virus 6	Unclassified	Unclassified	Unclassified	Unknown	MG256514	Partial genome	?	2018 (Tokarz et al., 2018)	*I. scapularis* (Tokarz et al., 2018)	USA (Tokarz et al., 2018)
Lone star tick associated virus 1	Unclassified	Unclassified	Unclassified	Unknown	MF962658	Partial genome	?	2018 (Tokarz et al., 2018)	*I. scapularis* (Tokarz et al., 2018)	USA (Tokarz et al., 2018)
Sapphire II virus	Unclassified	Unclassified	Unclassified	Unknown	MK896468, MK896467, MK896466	Complete genome	?	1972 (Yunker et al., 1972)	*A. cooleyi* (Yunker et al., 1972)	USA (Yunker et al., 1972)
Tick borne tetravirus‐like virus	Unclassified	Unclassified	Unclassified	Unknown	MW334982, KM048322	Partial genome	?	2014 (Tokarz et al., 2014)	*D. variabilis* (Tokarz et al., 2014)	USA (Tokarz et al., 2014)
Zirqa virus	Unclassified	Unclassified	Unclassified	Unknown	KU925500, KU925501, KU925502	Complete genome	?	1973 (Varma et al., 1973)	*A. cooleyi*, *Ornithodorus* spp. (Shi et al., 2018)	Abu Dhabi (Shi et al., 2018)
Bovine hepacivirus^b^	*Hepacivirus N*	*Hepacivirus*	Flaviviridae	ssRNA(+)	NC_026797	Complete genome	?	2015 (Baechlein et al., 2015)	Unknown (Temmam et al., 2019)	Brazil, China, Germany, Ghana, US (Baechlein et al., 2019)
HoBi‐like pestivirus^b^	*Pestivirus H*	*Pestivirus*	Flaviviridae	ssRNA(+)	MK689376	Partial genome	?	2004 (Schirrmeier et al., 2004)	Unknown (Temmam et al., 2019)	Argentina, Bangladesh, Brazil, China, Germany, India, Italy, Mexico, Thailand, Turkey, USA (Bauermann & Ridpath, 2021)
Erve virus^b^ ^,^ ^c^	*Erve orthonairovirus*	*Orthonairovirus*	Nairoviridae	ssRNA(−)	JF911697, JF911698, JF911699	Complete genome	?	1989 (Chastel et al., 1989)	Unknown (Dilcher et al., 2012)	Czech Republic, France, Germany, Netherland (Dilcher et al., 2012)
A “Bovine parvovirus‐2”‐like virus^b^	Unclassified	*Copiparvovirus*	Parvoviridae	ssDNA(+/−)	KT148961	Complete genome	?	2001 (Allander et al., 2001)	Unknown (Temmam et al., 2019)	Brazil, China, USA (Cibulski et al., 2016; Ng et al., 2015; Wang et al., 2018)
A “Nkolbisson virus”‐like virus^b^	Unclassified	*Ledantevirus*	Rhabdoviridae	ssRNA(−)	NC_034539	Complete genome	?	2010 (Dacheux et al., 2010)	Unknown (Temmam et al., 2019)	Cameroon (Dacheux et al., 2010)
A “Spring viremia of carp virus”‐like virus^b^	Unclassified	*Sprivivirus*	Rhabdoviridae	ssRNA(−)	NC_002803	Complete genome	?	1996 (Björklund et al., 1996)	Unknown (Temmam et al., 2019)	Austria, China, Czech Republic, Denmark, Hungary, Iran, Italy, Mexico, Moldova, Poland, Serbia, South Korea, Ukraine, UK, USA (Marsella et al., 2021)
A “Maraba virus”‐like virus^b^	Unclassified	*Vesiculovirus*	Rhabdoviridae	ssRNA(−)	NC_025255	Complete genome	?	2010 (Brun et al., 2010)	Unknown (Temmam et al., 2019)	Brazil (Brun et al., 2010)
A “Wellfleet Bay virus”‐like virus^b^	Unclassified	*Quaranjavirus*	Orthomyxoviridae	ssRNA(−)	NC_025793, NC_025794, NC_025795, NC_025796, NC_025797, NC_025798, NC_025799	Complete genome	?	2015 (Allison et al., 2015)	Unknown (Temmam et al., 2019)	USA (Allison et al., 2015)
Araguari virus^b^ ^,^ ^c^	Unclassified	*Thogotovirus*	Orthomyxoviridae	ssRNA(−)	KX670389, KX670390, KX670391, KX670392, KX670393, KX670394, KX670395	Complete genome	?	1969 (Da Silva et al., 2005)	Unknown (Da Silva et al., 2005)	Brazil (Da Silva et al., 2005)
An “Eelpout rhabdovirus”‐like virus^b^	Unclassified	Unclassified	Rhabdoviridae	ssRNA(−)	KR612230	Partial genome	?	2017 (Axén et al., 2016)	Unknown (Temmam et al., 2019)	Sweden (Axén et al., 2016)
A “Wuhan redfin culter dimarhabodovirus”‐like virus^b^	Unclassified	Unclassified	Rhabdoviridae	ssRNA(−)	MG600013	Complete genome	?	2018 (Shi et al., 2018)	Unknown (Temmam et al., 2019)	China (Shi et al., 2018)
Bat nairovirus/Ahun nairovirus^b^ ^,^ ^c^	Unclassified	*Nairovirus*	Nairoviridae	ssRNA(−)	KF170224	Partial genome	?	2014 (Dacheux et al., 2014)	Unknown (Dacheux et al., 2014)	France (Dacheux et al., 2014)
Essaouira virus^b^	Unclassified	Unclassified	Unclassified	Unknown	Not available	Not available	?	1993 (Chastel et al., 1993)	*O. maritimus* (Chastel et al., 1993)	Morocco (Chastel et al., 1993)
Huacho virus^b^	Unclassified	Unclassified	Unclassified	Unknown	Not available	Not available	?	1967 (Belaganahalli et al., 2015)	*C. amblus* (Belaganahalli et al., 2015)	Peru (Belaganahalli et al., 2015)
Kala Iris virus^b^	Unclassified	Unclassified	Unclassified	Unknown	Not available	Not available	?	1993 (Chastel et al., 1993)	*O. maritimus* (Chastel et al., 1993)	Morocco (Chastel et al., 1993)
Mono Lake virus^b^	Unclassified	Unclassified	Unclassified	Unknown	Not available	Not available	?	1988 (Calisher et al., 1988)	*A. cooleyi* (Calisher et al., 1988)	USA (Calisher et al., 1988)
Sixgun City virus^b^	Unclassified	Unclassified	Unclassified	Unknown	Not available	Not available	?	1988 (Calisher et al., 1988)	*A. cooleyi* (Calisher et al., 1988)	USA (Calisher et al., 1988)
Ellidaey virus^b^	Unclassified	Unclassified	Unclassified	Unknown	Not available	Not available	?	1986 (Moss et al., 1986)	*I. uriae* (Moss et al., 1986)	Iceland (Elliđaey island) (Moss et al., 1986)
Foula virus^b^	Unclassified	Unclassified	Unclassified	Unknown	Not available	Not available	?	1986 (Nuttall et al., 1986)	*I. uriae* (Nuttall et al., 1986)	Scotland (Shetland Islands) (Nuttall et al., 1986)
Grimsey virus^b^	Unclassified	Unclassified	Unclassified	Unknown	Not available	Not available	?	1986 (Moss et al., 1986)	*I. uriae* (Moss et al., 1986)	Iceland (Grimsey island) (Moss et al., 1986)
Inner Farne Island virus^b^	Unclassified	Unclassified	Unclassified	Unknown	Not available	Not available	?	1986 (Nuttall et al., 1986)	*I. uriae* (Nuttall et al., 1986)	England (Nuttall et al., 1986)
Mykines virus^b^	Unclassified	Unclassified	Unclassified	Unknown	Not available	Not available	?	1978 (Main, 1978)	*I. uriae* (Main, 1978)	Denmark (Faroe Islands) (Main, 1978)
Kao Shuan virus^b^	Unclassified	Unclassified	Unclassified	Unknown	Unavailable	Not available	?	1989 (Moussa & Main, 1989)	*A. robertsi* (Moussa & Main, 1989)	Indonesia, Taiwan (Moussa & Main, 1989)
Pretoria virus^b^	Unclassified	Unclassified	Unclassified	Unknown	Not available	Not available	?	1975 (Converse et al., 1975)	*A. africolumbae* (Converse et al., 1975)	South Africa (Converse et al., 1975)
Puffin Island virus^b^	Unclassified	Unclassified	Unclassified	Unknown	Not available	Not available	?	1983 (Gould et al., 1983)	*O. maritimus* (Gould et al., 1983)	Puffin Island (Gould et al., 1983)
Seletar virus^b^	Unclassified	Unclassified	Unclassified	Unknown	Not available	Not available	?	1961 (Shi et al., 2018)	*B. microplus* (Shi et al., 2018)	Malaysia, Singapore (Shi et al., 2018)

*Note*: ? means unknown.

Abbreviations: *A*., *Argas*; *Am*., *Amblyomma*; *B*., *Boophilus*; *D*., *Dermacentor*; *H*., *Haemaphysalis*; *Hy*., *Hyalomma*; *I*., *Ixodes*; *O*., *Ornithodoros*; *Ot*., *Otobius*; *R*., *Rhipicephalus*.

^a^ Amblyomma dissimile mivirus, Nuomin virus, and Granville quaranjavirus were later added tick‐associated viruses which were not included in the original tick virus database.

^b^ Viruses with unknown tick vectors or that sequences unavailable.

^c^ Tick infection unconfirmed but believed likely.

**TABLE A2:** Putative novel RNA viral contigs identified in this study.

Source data GenBank ID	Tick species	Provisional family	Genome structure	Sequence length (nt)	Closest BLASTn match (e‐value 0.005)	Sequence identity (%)	Predicted protein	References
GIXL01013119.1	*Ixodes ricinus*	Alphatetraviridae	ssRNA(+)	5496	Bulatov virus (MT025173.1)	78	One putative ORF; predicted protein contains homologues of RdRp 2 (PF00978) motif, nucleoside triphosphate hydrolase (SSF52540), viral (Superfamily 1) RNA helicase (PF01443) and viral methyltransferase 3 (PF01660) motifs	Trentelman et al. (2020), https://doi.org/10.1038/s41598‐020‐76268‐y
GBXQ01012426.1	*Ixodes persulcatus*	Alphatetraviridae	ssRNA(+)	1056	Vovk virus (MN830235.1)	81	One putative ORF; predicted protein contains homologues of RdRp 2 (PF00978) motif	X. C. Zhang, Unpublished.
GIXP02005464.1	*Ornithodoros moubata*	Alphatetraviridae	ssRNA(+)	5452	Bulatov virus (MT025173.1)	86	One putative ORF; predicted protein contains homologues of RdRp 2 (PF00978) motif, nucleoside triphosphate hydrolase (SSF52540), viral (Superfamily 1) RNA helicase (PF01443) and viral methyltransferase 3 (PF01660) motifs	Oleaga et al. (2021), https://doi.org/10.1371/journal.pntd.0009105
GFJQ02007921.1	*Ornithodoros moubata*	Alphatetraviridae	ssRNA(+)	3873	Bulatov virus (MT025173.1)	67	One putative ORF; predicted protein contains homologues of RdRp 2 (PF00978) motif, nucleoside triphosphate hydrolase (SSF52540) and viral (Superfamily 1) RNA helicase (PF01443) motifs	Oleaga et al. (2017), https://doi.org/10.1016/j.ttbdis.2017.05.002
GEMR01004364.1	*Rhipicephalus microplus*	Alphatetraviridae	ssRNA(+)	1488	*Hepelivirales* sp. (MW722078.1)	73	One putative ORF; predicted protein contains homologues of RdRp 2 (PF00978) motif.	Munoz et al. (2017), https://doi.org/10.1371/journal.pone.0172326
HACP01027211.1 (HACW01024387.1)	*Rhipicephalus sanguineus*	Orthomyxoviridae	ssRNA(−)	2136	Zambezi tick virus 1 (MH267793.1)	77	One putative ORF; predicted protein contains homologues of Influenza RdRp subunit PB1 (PF00602) motif	L. De Marco, S. Epis, F. Comandatore, D. Porretta, C. Cafarchia, F. Dantas‐Torres, D. Otranto, S. Urbanelli, C. Bandi, D. Sassera, and V. Mastrantonio, Unpublished.
HACP01022575.1	*Rhipicephalus sanguineus*	Orthomyxoviridae	ssRNA(−)	1290	Zambezi tick virus 1 (MH267793.1)	77	One putative ORF; predicted protein contains homologues of Influenza RdRp subunit PB1 (PF00602) motif	L. De Marco, S. Epis, F. Comandatore, D. Porretta, C. Cafarchia, F. Dantas‐Torres, D. Otranto, S. Urbanelli, C. Bandi, D. Sassera, and V. Mastrantonio, Unpublished.
HACW01018819.1	*Rhipicephalus sanguineus*	Orthomyxoviridae	ssRNA(−)	1290	Zambezi tick virus 1 (MH267793.1)	77	One putative ORF; predicted protein contains homologues of Influenza RdRp subunit PB1 (PF00602) motif	L. De Marco, S. Epis, F. Comandatore, D. Porretta, C. Cafarchia, F. Dantas‐Torres, D. Otranto, S. Urbanelli, C. Bandi, D. Sassera, and V. Mastrantonio, Unpublished.
GIJA01018702.1	*Rhipicephalus haemaphysaloides*	Orthomyxoviridae	ssRNA(−)	2357	Zambezi tick virus 1 (MH267793.1)	77	One putative ORF; predicted protein contains homologues of Influenza RdRp subunit PB1 (PF00602) motif	Wang, Wang, et al. (2020), https://doi.org/10.3389/fcimb.2020.00093
GGIX01201876.1	*Ixodes scapularis*	Chuviridae	ssRNA(−)	728	Bole tick virus 3 (MH688550.1)	87	One putative ORF; predicted protein contains homologues of Mononegavirales RdRp (PF00946) motif	H. M. Robertson, Unpublished.

**TABLE A3:** Defective RNA viral contigs identified in this study.

SRA project ID	Tick species	Provisional family	Genome structure	Sequence length (nt)	Closest BLASTn match (e‐value 0.005)	Sequence identity (%)	References
SRR10549473.54432.1	*Hy. asiaticum*	Chuviridae	ssRNA(−)	4383	Bole tick virus 3 (MH688550.1)	84	Yuan et al. (2020), https://doi.org/10.1016/j.jip.2020.107481
SRR10549473.681872.1	*Hy. asiaticum*	Chuviridae	ssRNA(−)	4528	Bole tick virus 3 (MH688550.1)	82	Yuan et al. (2020), https://doi.org/10.1016/j.jip.2020.107481
SRR10549473.681877.1	*Hy. asiaticum*	Chuviridae	ssRNA(−)	4420	Bole tick virus 3 (MH688550.1)	84	Yuan et al. (2020), https://doi.org/10.1016/j.jip.2020.107481
SRR10549473.681878.1	*Hy. asiaticum*	Chuviridae	ssRNA(−)	4440	Bole tick virus 3 (MH688550.1)	86	Yuan et al. (2020), https://doi.org/10.1016/j.jip.2020.107481
SRR10549473.779074.1	*Hy. asiaticum*	Chuviridae	ssRNA(−)	5286	Bole tick virus 3 (MH688550.1)	84	Yuan et al. (2020), https://doi.org/10.1016/j.jip.2020.107481
SRR10549473.779079.1	*Hy. asiaticum*	Chuviridae	ssRNA(−)	5379	Bole tick virus 3 (MH688550.1)	85	Yuan et al. (2020), https://doi.org/10.1016/j.jip.2020.107481
SRR10549473.779081.1	*Hy. asiaticum*	Chuviridae	ssRNA(−)	5334	Bole tick virus 3 (MH688550.1)	83	Yuan et al. (2020), https://doi.org/10.1016/j.jip.2020.107481
SRR10549473.1447289.1	*Hy. asiaticum*	Chuviridae	ssRNA(−)	5272	Bole tick virus 3 (MH688550.1)	85	Yuan et al. (2020), https://doi.org/10.1016/j.jip.2020.107481
SRR10549473.1447293.1	*Hy. asiaticum*	Chuviridae	ssRNA(−)	5001	Bole tick virus 3 (MH688550.1)	87	Yuan et al. (2020), https://doi.org/10.1016/j.jip.2020.107481
SRR10549473.2865317.1	*Hy. asiaticum*	Chuviridae	ssRNA(−)	5556	Bole tick virus 3 (MH688550.1)	83	Yuan et al. (2020), https://doi.org/10.1016/j.jip.2020.107481
SRR10549473.2865320.1	*Hy. asiaticum*	Chuviridae	ssRNA(−)	5643	Bole tick virus 3 (MH688550.1)	87	Yuan et al. (2020), https://doi.org/10.1016/j.jip.2020.107481
SRR10549473.4598504.1	*Hy. asiaticum*	Chuviridae	ssRNA(−)	8170	Bole tick virus 3 (MH688550.1)	88	Yuan et al. (2020), https://doi.org/10.1016/j.jip.2020.107481
SRR10549473.4598510.1	*Hy. asiaticum*	Chuviridae	ssRNA(−)	8206	Bole tick virus 3 (MH688550.1)	87	Yuan et al. (2020), https://doi.org/10.1016/j.jip.2020.107481
SRR10549473.4598511.1	*Hy. asiaticum*	Chuviridae	ssRNA(−)	8182	Bole tick virus 3 (MH688550.1)	85	Yuan et al. (2020), https://doi.org/10.1016/j.jip.2020.107481
SRR10549473.7000060.1	*Hy. asiaticum*	Chuviridae	ssRNA(−)	4986	Bole tick virus 3 (MH688550.1)	84	Yuan et al. (2020), https://doi.org/10.1016/j.jip.2020.107481
SRR10549473.10341027.1	*Hy. asiaticum*	Chuviridae	ssRNA(−)	2448	Bole tick virus 3 (MH688550.1)	86	Yuan et al. (2020), https://doi.org/10.1016/j.jip.2020.107481
SRR10549473.10526706.1	*Hy. asiaticum*	Chuviridae	ssRNA(−)	6273	Bole tick virus 3 (MH688550.1)	87	Yuan et al. (2020), https://doi.org/10.1016/j.jip.2020.107481
SRR10549473.10526708.1	*Hy. asiaticum*	Chuviridae	ssRNA(−)	6276	Nuomin virus (MW029964.1)	78	Yuan et al. (2020), https://doi.org/10.1016/j.jip.2020.107481
SRR17281102.635386.1	*I. scapualris*	Chuviridae	ssRNA(−)	1390	Suffolk virus (NC_028243.1)	78	University of Minnesota, Unpublished.

Abbreviations: *Hy.*, *Hyalomma*; *I.*, *Ixodes*.

**TABLE A4:** DNA viral contigs identified in this study.

WGS project ID	Tick species	Genome structure	Sequence length (bp)	Predicted protein	References
GIXP02029293.1	*Ornithodoros moubata*	dsDNA	2232	DdRp subunit 3 (encoded by *H359L*); ATP‐dependent RNA helicase (encoded by *Q706L*); ATP‐dependent RNA helicase (encoded by *QP509L*)	Oleaga et al. (2021), https://doi.org/10.1371/journal.pntd.0009105
GIXP02006837.1	*Ornithodoros moubata*	dsDNA	1450	DdRp subunit 3 (encoded by *H359L*); Uncharacterised protein (encoded by *H171R)*	Oleaga et al. (2021), https://doi.org/10.1371/journal.pntd.0009105
CAJHNL010000002.1	*Ornithodoros porcinus*	dsDNA	7421	DdRp subunit 2 (encoded by *EP1242L*); Helicase (encoded by *F1055L*)	Forth et al. (2020), https://doi.org/10.1186/s12915‐020‐00865‐6
CAJHNL010000003.1	*Ornithodoros porcinus*	dsDNA	6757	Transcription factor SII homologue (encoded by *I243L*); pI226R; RPB7 homologue; Uncharacterised protein (encoded by *D339L)*; Uncharacterised protein (encoded by *D129L*)	Forth et al. (2020), https://doi.org/10.1186/s12915‐020‐00865‐6
CAJHNL010000004.1	*Ornithodoros porcinus*	dsDNA	6577	Primase (encoded by *C962R*)	Forth et al. (2020), https://doi.org/10.1186/s12915‐020‐00865‐6
CAJHNL010000005.1	*Ornithodoros porcinus*	dsDNA	3455	TATA‐binding protein (encoded by *B263R*)	Forth et al. (2020), https://doi.org/10.1186/s12915‐020‐00865‐6
CAJHNL010000006.1	*Ornithodoros porcinus*	dsDNA	3089	DdRp subunit 2 (encoded by *EP1242L*)	Forth et al. (2020), https://doi.org/10.1186/s12915‐020‐00865‐6
CAJHNL010000007.1	*Ornithodoros porcinus*	dsDNA	1557	DdDp (encoded by *G1211R*)	Forth et al. (2020), https://doi.org/10.1186/s12915‐020‐00865‐6
CAJHNL010000008.1	*Ornithodoros porcinus*	dsDNA	629	Ribonucleotide reductase large subunit (encoded by *F778R*); Uncharacterised protein p360 15R	Forth et al. (2020), https://doi.org/10.1186/s12915‐020‐00865‐6
CAJHNP010000002.1	*Ornithodoros moubata*	dsDNA	7383	DdRp subunit 2 (encoded by *EP1242L*); Ribonucleotide reductase large subunit (encoded by *F778R*); Helicase (encoded by *F1055L*)	Forth et al. (2020), https://doi.org/10.1186/s12915‐020‐00865‐6
CAJHNP010000004.1	*Ornithodoros moubata*	dsDNA	670	DdRp subunit 3 (encoded by *H359L*)	Forth et al. (2020), https://doi.org/10.1186/s12915‐020‐00865‐6
CAJHNP010000005.1	*Ornithodoros moubata*	dsDNA	5156	Viral histone‐like protein (encoded by *A104R*); Inhibitor of apoptosis protein (encoded by *A224L*); Thymidylate kinase (encoded by *A240L*); Uncharacterised protein (encoded by *A151R*)	Forth et al. (2020), https://doi.org/10.1186/s12915‐020‐00865‐6
CAJHNP010000006.1	*Ornithodoros moubata*	dsDNA	3634	DdRp subunit 3 (encoded by *H359L*); Uncharacterised protein *CP123L*; ATP‐dependent RNA helicase (encoded by *QP509L*); DNA topoisomerase 2 (encoded by *P1192R*)	Forth et al. (2020), https://doi.org/10.1186/s12915‐020‐00865‐6
CAJHNP010000007.1	*Ornithodoros moubata*	dsDNA	2713	Early transcription factor large subunit (encoded by *D1133L*); Uncharacterised protein (encoded by *H124R*); Uncharacterised protein (encoded by *H171R*)	Forth et al. (2020), https://doi.org/10.1186/s12915‐020‐00865‐6
CAJHNP010000017.1	*Ornithodoros moubata*	dsDNA	1007	Primase (encoded by *C962R*)	Forth et al. (2020), https://doi.org/10.1186/s12915‐020‐00865‐6
CAJHNQ010000010.1	*Ornithodoros moubata*	dsDNA	636	DdRp subunit 2 (encoded by *EP1242L*)	Forth et al. (2020), https://doi.org/10.1186/s12915‐020‐00865‐6
CAJHNQ010000014.1	*Ornithodoros moubata*	dsDNA	679	DdRp subunit 2 (encoded by *EP1242L*); Ribonucleotide reductase large subunit (encoded by *F778R*); Helicase (encoded by *F1055L*)	Forth et al. (2020), https://doi.org/10.1186/s12915‐020‐00865‐6
CAJHNQ010000015.1	*Ornithodoros moubata*	dsDNA	1193	Helicase (encoded by *F1055L*)	Forth et al. (2020), https://doi.org/10.1186/s12915‐020‐00865‐6
CAJHNQ010000016.1	*Ornithodoros moubata*	dsDNA	1697	DdRp subunit 2 (encoded by *EP1242L*); Helicase (encoded by *F1055L*)	Forth et al. (2020), https://doi.org/10.1186/s12915‐020‐00865‐6
CAJHNR010000004.1	*Ornithodoros moubata*	dsDNA	16,841	DdRp subunit 2 (encoded by *EP1242L*); Ribonucleotide reductase large subunit (encoded by *F778R*); Helicase (encoded by *F1055L*)	Forth et al. (2020), https://doi.org/10.1186/s12915‐020‐00865‐6
CAJHNR010000005.1	*Ornithodoros moubata*	dsDNA	15,286	TATA‐binding protein (encoded by *B263R*)	Forth et al. (2020), https://doi.org/10.1186/s12915‐020‐00865‐6
CAJHNR010000006.1	*Ornithodoros moubata*	dsDNA	13,138	TATA‐binding protein (encoded by *B263R*)	Forth et al. (2020), https://doi.org/10.1186/s12915‐020‐00865‐6
CAJHNR010000011.1	*Ornithodoros moubata*	dsDNA	8352	Helicase (encoded by *A859L*); Uncharacterised protein p360 15R; Uncharacterised protein 5ELp28; IkB‐like protein (encoded by *A238L*)	Forth et al. (2020), https://doi.org/10.1186/s12915‐020‐00865‐6
CAJHNR010000013.1	*Ornithodoros moubata*	dsDNA	7718	Early transcription factor large subunit (encoded by *D1133L*); DdRp subunit 3 (encoded by *H359L*); Uncharacterised protein H171R	Forth et al. (2020), https://doi.org/10.1186/s12915‐020‐00865‐6
CAJHNR010000014.1	*Ornithodoros moubata*	dsDNA	7045	TATA‐binding protein (encoded by *B263R*)	Forth et al. (2020), https://doi.org/10.1186/s12915‐020‐00865‐6
CAJHNR010000015.1	*Ornithodoros moubata*	dsDNA	6370	TATA‐binding protein (encoded by *B263R*); Early transcription factor large subunit (encoded by *G1340L*); Primase (encoded by *C962R*)	Forth et al. (2020), https://doi.org/10.1186/s12915‐020‐00865‐6
CAJHNR010000016.1	*Ornithodoros moubata*	dsDNA	5808	TATA‐binding protein (encoded by *B263R*)	Forth et al. (2020), https://doi.org/10.1186/s12915‐020‐00865‐6
CAJHNR010000017.1	*Ornithodoros moubata*	dsDNA	5688	TATA‐binding protein (encoded by *B263R*)	Forth et al. (2020), https://doi.org/10.1186/s12915‐020‐00865‐6
CAJHNR010000018.1	*Ornithodoros moubata*	dsDNA	5489	DdRp subunit 3 (encoded by *H359L*); Uncharacterised protein (encoded by *CP123L*); DNA topoisomerase II (encoded by *P1192R*)	Forth et al. (2020), https://doi.org/10.1186/s12915‐020‐00865‐6
CAJHNR010000019.1	*Ornithodoros moubata*	dsDNA	5396	Uncharacterised protein p360 15R	Forth et al. (2020), https://doi.org/10.1186/s12915‐020‐00865‐6
CAJHNR010000020.1	*Ornithodoros moubata*	dsDNA	5152	Histone‐like protein (encoded by *A104R*); Inhibitor of apoptosis protein (encoded by *A224L*); Thymidylate kinase (encoded by *A240L*); Uncharacterised protein (encoded by *A151R*)	Forth et al. (2020), https://doi.org/10.1186/s12915‐020‐00865‐6
CAJHNR010000021.1	*Ornithodoros moubata*	dsDNA	4920	DdRp subunit 10 (encoded by *CP80R*); Uncharacterised protein (encoded by *CP312R*)	Forth et al. (2020), https://doi.org/10.1186/s12915‐020‐00865‐6
CAJHNR010000023.1	*Ornithodoros moubata*	dsDNA	4152	Primase (encoded by *C962R*)	Forth et al. (2020), https://doi.org/10.1186/s12915‐020‐00865‐6
CAJHNR010000024.1	*Ornithodoros moubata*	dsDNA	3824	DdRp subunit 2 (encoded by *EP1242L*); Transmembrane protein (encoded by *EP84R*)	Forth et al. (2020), https://doi.org/10.1186/s12915‐020‐00865‐6
CAJHNR010000025.1	*Ornithodoros moubata*	dsDNA	3339	DdRp subunit 2 (encoded by *EP1242L*)	Forth et al. (2020), https://doi.org/10.1186/s12915‐020‐00865‐6
CAJHNR010000026.1	*Ornithodoros moubata*	dsDNA	2712	DdRp subunit 2 (encoded by *EP1242L*)	Forth et al. (2020), https://doi.org/10.1186/s12915‐020‐00865‐6
CAJHNR010000027.1	*Ornithodoros moubata*	dsDNA	2653	Putative poly(A) polymerase catalytic subunit (encoded by *C475L*); Primase (encoded by *C962R*)	Forth et al. (2020), https://doi.org/10.1186/s12915‐020‐00865‐6
CAJHNR010000028.1	*Ornithodoros moubata*	dsDNA	2475	DdRp subunit 3 (encoded by *H359L*)	Forth et al. (2020), https://doi.org/10.1186/s12915‐020‐00865‐6
CAJHNR010000029.1	*Ornithodoros moubata*	dsDNA	2196	Ribonucleotide reductase large subunit (encoded by *F778R*)	Forth et al. (2020), https://doi.org/10.1186/s12915‐020‐00865‐6
CAJHNR010000030.1	*Ornithodoros moubata*	dsDNA	2179	Uncharacterised protein (encoded by CP123L); mRNA‐capping enzyme (encoded by *NP868R*)	Forth et al. (2020), https://doi.org/10.1186/s12915‐020‐00865‐6
CAJHNR010000032.1	*Ornithodoros moubata*	dsDNA	1763	Ribonucleotide reductase large subunit (encoded by *F778R*)	Forth et al. (2020), https://doi.org/10.1186/s12915‐020‐00865‐6
CAJHNS010000026.1	*Ornithodoros moubata*	dsDNA	1078	Helicase (encoded by *F1055L*)	Forth et al. (2020), https://doi.org/10.1186/s12915‐020‐00865‐6
CAJHNU010000002.1	*Ornithodoros porcinus*	dsDNA	3380	DdRp subunit 2 (encoded by *EP1242L*); Helicase (encoded by *F1055L*)	Forth et al. (2020), https://doi.org/10.1186/s12915‐020‐00865‐6
CAJHNU010000003.1	*Ornithodoros porcinus*	dsDNA	641	Ribonucleotide reductase large subunit (encoded by *F778R*)	Forth et al. (2020), https://doi.org/10.1186/s12915‐020‐00865‐6
CAJHNU010000004.1	*Ornithodoros porcinus*	dsDNA	1803	TATA‐binding protein (encoded by *B263R*)	Forth et al. (2020), https://doi.org/10.1186/s12915‐020‐00865‐6
CAJHNU010000009.1	*Ornithodoros porcinus*	dsDNA	776	Transcription factor S‐II‐related protein (encoded by *I243L*); Late protein (encoded by *I226R*)	Forth et al. (2020), https://doi.org/10.1186/s12915‐020‐00865‐6
CAJHNU010000011.1	*Ornithodoros porcinus*	dsDNA	2551	Primase (encoded by *C962R*)	Forth et al. (2020), https://doi.org/10.1186/s12915‐020‐00865‐6
CAJHNU010000014.1	*Ornithodoros porcinus*	dsDNA	3100	DdRp subunit 2 (encoded by *EP1242L*)	Forth et al. (2020), https://doi.org/10.1186/s12915‐020‐00865‐6
CAJHNV010000002.1	*Ornithodoros porcinus*	dsDNA	4758	DdRp subunit 2 (encoded by *EP1242L*)	Forth et al. (2020), https://doi.org/10.1186/s12915‐020‐00865‐6
CAJHNV010000004.1	*Ornithodoros porcinus*	dsDNA	3378	Transcription factor SII homologue (encoded by *I243L*); Late protein (encoded by *I226R*); Uncharacterised protein (encoded by *D79L*)	Forth et al. (2020), https://doi.org/10.1186/s12915‐020‐00865‐6
CAJHNV010000005.1	*Ornithodoros porcinus*	dsDNA	2605	DdRp subunit 2 (encoded by *EP1242L*)	Forth et al. (2020), https://doi.org/10.1186/s12915‐020‐00865‐6
CAJHNV010000007.1	*Ornithodoros porcinus*	dsDNA	777	Transcription factor SII homologue (encoded by *I243L*)	Forth et al. (2020), https://doi.org/10.1186/s12915‐020‐00865‐6
CAJHNW010000016.1	*Ornithodoros moubata*	dsDNA	1078	Helicase (encoded by *F1055L*)	Forth et al. (2020), https://doi.org/10.1186/s12915‐020‐00865‐6

**TABLE A5:** GenBank ID of viruses phylogenetically related to the putative novel tick viruses. Zoonotic risks of virus sequences (in GenBank format) were predicted using the GCB model.

	Viral species	GenBank ID
Relative viral species of putative tick alphatetraviruses	*Nudaurelia capensis beta virus*	NC_001990.1
		NC_005898.1; NC_005899.1
	*Helicoverpa armigera stunt virus*	NC_001981.1; NC_001982.1
Relative viral species of putative tick orthomyxoviruses	*Wellfleet Bay virus*	NC_025798.1; NC_025797.1; NC_025796.1; NC_025795.1; NC_025794.1; NC_025793.1; NC_025799.1
	*Araguari virus*	KX670389.1; KX670390.1; KX670391.1; KX670392.1; KX670393.1; KX670394.1; KX670395.1
	*Johnston Atoll quaranjavirus*	NC_052931.1; NC_052930.1; NC_052929.1; NC_052928.1; NC_052927.1; NC_052926.1; NC_052925.1
	*Lake Chad virus*	NC_052682.1; NC_052686.1; NC_052685.1; NC_052684.1; NC_052683.1; NC_052681.1; NC_052680.1
	*Tjuloc virus*	JQ928941.1; JQ928942.1; JQ928943.1; JQ928944.1; JQ928945.1; JQ928946.1; MT774252.1
	*Quaranfil quaranjavirus*	NC_038821.1; NC_038822.1; NC_038820.1; NC_038819.1; NC_038818.1; NC_038817.1
Relative viral species of putative tick chuviruses	*Mivirus suffolkense*	NC_028243

**TABLE A6:** Zoonotic ranking of 133 known tick‐associated viruses with complete genomes. Zoonotic risks of virus sequences (in GenBank format) were predicted using the GCB model.

Virus name	Acronym	Human infection	Predicted probability mean	Predicted probability lower	Predicted probability upper	Rank	Priority
Tick‐borne encephalitis virus	TBEV	Y (Shi et al., 2018)	.777	.446	.946	1	Very high
Omsk haemorrhagic fever virus	OHFV	Y (Grard et al., 2007)	.739	.443	.951	2	Very high
Powassan virus	POWV	Y (Tokarz et al., 2014)	.695	.288	.920	3	High
Isfahan virus	ISFV	?	.677	.233	.917	4	High
Uukuniemi virus	UUKV	?	.676	.262	.944	5	High
Louping ill virus	LIV	Y (Davidson et al., 1991)	.668	.288	.890	6	High
Langat virus	LGTV	?	.667	.339	.917	7	Very high
Thogoto virus	THOV	Y (Kosoy et al., 2015)	.642	.266	.899	8	High
Lonestar tick chuvirus 1	LSTCV‐1	?	.619	.379	.857	9	Very high
Crimean‐Congo hemorrhagic fever virus	CCHFV	Y (Garrison et al., 2020)	.609	.249	.875	10	High
Grotenhout virus	GROTHV	?	.601	.389	.808	11	Very high
Severe fever with thrombocytopenia syndrome virus	SFTSV	Y (Yu et al., 2011)	.596	.273	.849	12	High
Kasokero virus	KASV	?	.580	.180	.837	13	High
Colorado tick fever virus	CTFV	Y (Shi et al., 2018)	.562	.121	.854	14	High
Quaranfil virus	QRFV	Y (Mourya et al., 2019)	.561	.128	.863	15	High
Great Island virus	GIV	?	.550	.115	.832	16	High
Dugbe virus	DUGV	Y (Garrison et al., 2020)	.540	.157	.825	17	High
Johnston Atoll virus	JAV	?	.513	.311	.802	18	Very high
Dabieshan tick virus	DBSH	?	.505	.289	.747	19	High
Taggert virus	TAGV	?	.503	.318	.802	20	Very high
Blacklegged tick phlebovirus 1	BLTPV‐1	?	.490	.271	.711	21	High
Deer tick virus	DTV	?	.486	.275	.703	22	High
Tacheng tick virus 2	TcTV‐2	Y (Dong et al., 2021)	.482	.241	.774	23	High
Lihan tick virus	LhTV	?	.481	.264	.730	24	High
Karshi virus	KSIV	?	.481	.266	.954	25	High
Alkhumra haemorrhagic fever virus	AHFV	Y (Grard et al., 2007)	.469	.234	.736	26	High
Yongjia tick virus 1	YjTV‐1	?	.441	.192	.680	27	High
American dog tick phlebovirus	ADTPV	?	.438	.218	.659	28	High
Wenzhou tick virus	WzTV	?	.425	.234	.710	29	High
Tamdy virus	TAMV	Y (Moming et al., 2021)	.413	.227	.642	30	High
Blacklegged tick rhabdovirus 1	BTRV‐1	?	.411	.223	.638	31	High
Tacheng tick virus 8	TcTV‐8	?	.404	.210	.692	32	High
Jingmen tick virus	JMTV	Y (Jia et al., 2019)	.401	.219	.663	33	High
South Bay virus	SBV	?	.395	.210	.630	34	High
Ngari virus	NRI	Y (Groseth et al., 2012)	.393	.214	.624	35	High
Kaisodi virus	KSDV	?	.392	.208	.590	36	High
Kupe virus	KUPEV	?	.391	.160	.642	37	High
Lone star tick densovirus 1	LSTDV‐1	?	.391	.221	.654	38	High
Blacklegged tick phlebovirus 3	BLTPV‐3	?	.371	.195	.640	39	High
Pacific coast tick nairovirus	PCTNV	?	.370	.197	.656	40	High
Clo Mor virus	CLMV	?	.364	.149	.600	41	High
Sakhalin virus	SAKV	?	.345	.177	.657	42	High
Tyuleniy virus	TYUV	?	.340	.160	.739	43	High
Meram virus	MERV	?	.335	.196	.574	44	High
Jos virus	JOSV	?	.333	.159	.579	45	High
Midway virus	MIDWV	?	.327	.163	.609	46	High
Lone star virus	LSV	?	.326	.166	.567	47	High
Norway phlebovirus 1	NPV‐1	?	.323	.138	.559	48	High
Tofla virus	TFLV	?	.323	.198	.601	49	High
Tacheng tick virus 7	TcTV‐7	?	.318	.175	.511	50	High
New Minto virus	NMV	?	.318	.168	.526	51	High
Bourbon virus	BRBV	Y (Kosoy et al., 2015)	.311	.164	.497	52	High
Suffolk virus	SUFV	?	.309	.151	.562	53	High
Silverwater virus	SWV	?	.307	.156	.537	54	High
Heartland virus	HRTV	Y (McMullan et al., 2012)	.305	.135	.520	55	High
Amblyomma dissimile mivirus	ADM	?	.301	.146	.511	56	High
Dera Ghazi Khan virus	DGKV	?	.299	.150	.493	57	High
Hunter Island virus	HIGV	?	.299	.153	.528	58	High
Great Saltee virus	GRSV	?	.299	.168	.543	59	High
Bhanja virus	BHAV	Y (Matsuno et al., 2013)	.298	.161	.446	60	High
Blanchseco virus	BCOV	?	.295	.153	.500	61	High
Thailand tick thogotovirus	TT‐THOV	?	.286	.135	.436	62	Medium
Wuhan tick virus 1	WhTV‐1	?	.280	.145	.461	63	Medium
Gadgets Gully virus	GGYV	?	.277	.152	.583	64	Medium
Soldado virus	SOLV	?	.277	.164	.450	65	Medium
Abu Hammad virus	AHV	?	.275	.138	.471	66	Medium
Kadam virus	KADV	?	.275	.134	.602	67	Medium
Tacheng tick virus 1	TcTV‐1	Y (Liu et al., 2020)	.272	.134	.514	68	Medium
Zirqa virus	ZIRV	?	.269	.146	.439	69	Medium
Bole tick virus 2	BLTV‐2	?	.267	.141	.466	70	Medium
Qalyub virus	QYBV	?	.264	.112	.444	71	Medium
Avalon virus	AVAV	?	.259	.119	.449	72	Medium
Genoa virus	GEV	?	.259	.131	.430	73	Medium
Upolu virus	UPOV	?	.258	.130	.419	74	Medium
Blacklegged tick chuvirus 2	BLTCV‐2	?	.258	.137	.453	75	Medium
Long Island tick rhabdovirus	LITRV	?	.257	.118	.473	76	Medium
Taishun tick virus	TsTV	?	.256	.131	.394	77	Medium
Kemerovo virus	KEMV	?	.255	.100	.425	78	Medium
Wuhan tick virus 2	WhTV‐2	?	.253	.133	.460	79	Medium
Raza virus	RAZAV	?	.250	.139	.432	80	Medium
Tillamook virus	TILLV	?	.248	.118	.495	81	Medium
New Kent County virus	NKCV	?	.246	.132	.359	82	Medium
Issyk‐Kul virus	ISKV	?	.235	.105	.343	83	Medium
Zahedan rhabdovirus	ZARV	?	.234	.119	.386	84	Medium
Tacheng tick virus 3	TcTV‐3	?	.232	.106	.382	85	Medium
Tunis virus	TUNV	?	.232	.105	.369	86	Medium
Tacheng tick virus 5	TcTV‐5	?	.232	.131	.359	87	Medium
Sawgrass virus	SAWV	?	.231	.122	.403	88	Medium
Changping tick virus 2	CpTV‐2	?	.226	.111	.410	89	Medium
Nyamanini virus	NYMV	?	.222	.123	.394	90	Medium
Dhori virus	DHOV	Y (Kosoy et al., 2015)	.222	.114	.498	91	Medium
Vinegar Hill virus	VINHV	?	.221	.118	.367	92	Medium
Huangpi tick virus 3	HpTV‐3	?	.217	.106	.347	93	Medium
Huangpi tick virus 1	HpTV‐1	?	.216	.102	.399	94	Medium
Meaban virus	MEAV	?	.216	.100	.445	95	Medium
Hazara virus	HAZV	?	.216	.125	.419	96	Medium
Punta Salinas virus	PSV	?	.212	.102	.433	97	Medium
Wuhan mivirus	WhMV	?	.212	.108	.382	98	Medium
Laurel Lake virus	LLV	?	.209	.085	.324	99	Medium
Canine circovirus	CaCV	?	.208	.101	.361	100	Medium
Matruh virus	MTRV	?	.203	.111	.311	101	Medium
Connecticut virus	CNTV	?	.201	.098	.376	102	Medium
Sapphire II virus	SAPV	?	.197	.098	.319	103	Medium
Tacheng tick virus 6	TcTV‐6	?	.197	.093	.368	104	Medium
Changping tick virus 3	CpTV‐3	?	.195	.085	.331	105	Medium
Saumarez Reef virus	SREV	?	.194	.098	.371	106	Medium
Abu Mina virus	AMV	?	.193	.101	.355	107	Medium
Tarumizu tick virus	TarTV	?	.192	.104	.339	108	Medium
Eyach virus	EYAV	?	.192	.103	.362	109	Medium
Barur virus	BARV	?	.191	.091	.347	110	Medium
Bahig virus	BAHV	?	.190	.092	.324	111	Medium
Sierra Nevada virus	SNVV	?	.186	.080	.329	112	Medium
Wad Medani virus	WMV	?	.186	.105	.401	113	Medium
Kundal virus	KUNDV	?	.184	.095	.312	114	Medium
Chobar Gorge virus	CGV	?	.178	.096	.337	115	Medium
African swine fever virus	ASFV	?	.175	.082	.311	116	Medium
Farallon virus	FARV	?	.175	.088	.312	117	Medium
Oz virus	OZV	?	.174	.089	.283	118	Low
Nairobi sheep disease virus	NSDV	Y (Yadav et al., 2011)	.173	.091	.324	119	Medium
Keterah virus	KETV	?	.169	.089	.314	120	Medium
Estero Real virus	ERV	?	.167	.087	.315	121	Medium
Geran virus	GERV	?	.165	.082	.282	122	Low
Bole tick virus 4	BLTV‐4	?	.164	.081	.315	123	Medium
Caspiy virus	CASV	?	.164	.081	.281	124	Low
Hughes virus	HUGV	?	.161	.085	.267	125	Low
Tacheng tick virus 4	TcTV‐4	?	.158	.071	.257	126	Low
St Croix River virus	SCRV	?	.153	.076	.264	127	Low
Bandia virus	BDAV	?	.148	.077	.245	128	Low
Chenuda virus	CNUV	?	.146	.079	.226	129	Low
Kolente virus	KOLEV	?	.142	.072	.241	130	Low
Yongjia tick virus 2	YjTV‐2	?	.132	.057	.219	131	Low
Bole tick virus 3	BLTV‐3	?	.131	.051	.231	132	Low
Avian‐like circovirus	ALCV	?	.126	.056	.216	133	Low

*Note*: ? means unknown.
